# Neutrophil degranulation in the lung microenvironment linked to idiopathic pulmonary fibrosis severity and survival

**DOI:** 10.1016/j.isci.2025.114125

**Published:** 2025-11-19

**Authors:** Scott M. Matson, Linh T. Ngo, Yui Sugawara, Veani Fernando, Claudia Lugo, Angela Kaczorowski-Worthley, Imaan Azeem, Alexis Harrison, Alex Alsup, Emily Schueddig, Devin Koestler, Michaella J. Rekowski, Paul J. Wolters, Joyce S. Lee, Michael P. Washburn, Joshua J. Solomon, M. Kristen Demoruelle

**Affiliations:** 1 Division of Pulmonary, Critical Care and Sleep Medicine, University of Kansas School of Medicine, Kansas City, KS, USA; 2 Department of Respiratory Medicine, Respiratory Center, Toranomon Hospital, Tokyo, Japan; 3 Division of Rheumatology, University of Colorado School of Medicine, Aurora, CO, USA; 4 School of Medicine, University of Kansas, Kansas City, KS, USA; 5 Department of Biostatistics & Data Science, University of Kansas School of Medicine, Kansas City, KS, USA; 6 Department of Cancer Biology, University of Kansas School of Medicine, Kansas City, KS, USA; 7 Division of Pulmonary and Critical Care Medicine, University of California, San Francisco, San Francisco, CA, USA; 8 Division of Pulmonary Sciences and Critical Care Medicine, University of Colorado, Aurora, CO, USA; 9 Division of Pulmonary, Critical Care and Sleep Medicine, National Jewish Health Hospital, Denver, CO, USA

**Keywords:** Fibrosis, Body substance sample, Components of the immune system, Proteomics

## Abstract

Idiopathic pulmonary fibrosis (IPF) causes progressive respiratory failure with variable survival trajectories among patients. Neutrophils accumulate in IPF lungs, but their mechanistic contribution to disease progression remains to be determined. We applied label-free quantitative proteomics to IPF lung tissue (*n* = 10) and bronchoalveolar lavage fluid (BALF) (*n* = 50) from patients with distinct survival outcomes. Neutrophil degranulation emerged as the pathway most strongly associated with poor survival in lung tissue and second most significant in BALF. We validated these findings using absolute quantification of neutrophil degranulation markers in two independent IPF cohorts (*n* = 156 and *n* = 52). Higher BALF levels of extracellular DNA, DNA-myeloperoxidase complexes, calprotectin, and neutrophil elastase predicted worse survival (hazard ratios: 1.79–2.19) and correlated with reduced lung function. These results identify neutrophil degranulation as a compartment-specific mechanism of lung injury in IPF that may guide therapeutic development and risk stratification.

## Highlights

• Proteomics links neutrophil degranulation to worse survival in lung fibrosis
• Bronchoalveolar markers predict poor survival independent of clinical scores
• Bronchoalveolar markers associate with lower lung function across cohorts
• Blood markers were not predictive, highlighting lung-specific activity

## Graphical abstract

**Figure:**
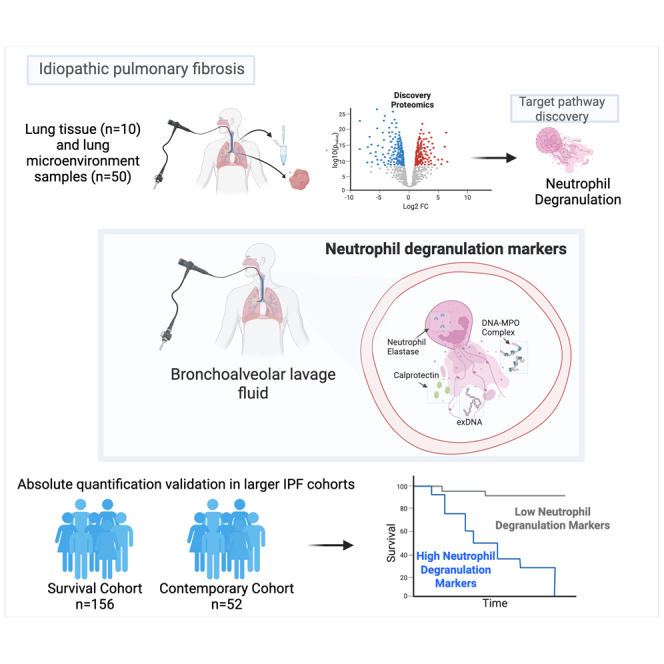

## Introduction

Idiopathic pulmonary fibrosis (IPF) is a devastating and universally progressive condition with survival of 3–5 years from the time of diagnosis.^1^ Current treatment options with antifibrotic therapy have been shown to slow lung function decline compared to placebo but fail to improve survival.^2^^,^^3^ Given the limitations of currently available therapies and the grim prognosis of IPF, the discovery of novel mechanisms of lung injury and targetable endotypes of IPF could offer new therapeutic targets.

Agnostic, systems-biology-based methods, such as label-free quantitative proteomics, have been explored in IPF with a focus to identify proteomic signatures that distinguish IPF from normal control populations.^4^^,^^5^^,^^6^^,^^7^^,^^8^^,^^9^^,^^10^ These findings have led to important insights into IPF pathobiology, but key features associated with survival in IPF remain elusive. Despite the median survival rate being dismal, there exists a spectrum of survival across IPF patients. Therefore, we leveraged IPF patients with distinct survival trajectories and applied a shotgun, agnostic proteomic method in lung tissue and the lung microenvironment via bronchoalveolar lavage fluid (BALF) to compare differential protein expression and pathways that could identify novel candidate targets associated with poor IPF survival. We then confirmed our discovery proteomics findings in the IPF lung microenvironment via absolute quantification of pathway-associated protein targets in BALF from two independent IPF cohorts and assessed the clinical impact of these pathway-associated markers.

## Results

### Patient characteristics

In the lung tissue discovery proteomics cohort (*n* = 10), the mean (SD) days from tissue collection to death was 143.2 (89.5) for the short survival group and 1,394.8 (881.2) for the long survival group (mean difference of 1,251.6 days, *p* < 0.01). Age, FVC%, and DLCO% were similar across the two groups, although the short survival group had a higher proportion of patients on immunosuppression. Most patients in the cohort were male with a history of smoking (Table 1).

**Table 1:** IPF lung tissue discovery proteomics cohort

	Short survival (*n* = 5)	Long survival (*n* = 5)	P-value
Age, mean (SD)	63.0 (10.7)	61.6 (6.1)	0.81
Gender, n (%) male	4 (80%)	2 (40%)	0.41
Smoker, n (%) ever	4 (80%)	2 (40%)	0.41
FVC% predicted, mean (SD)	50.6 (13.5)	67.0 (12.9)	0.086
DLCO% predicted, mean (SD)	73.6 (21.3)	92.5 (6.8)	0.14
On immunosuppression, n (%)	2 (40%)	0 (0%)	0.44
Survived to 5 year, %	0 (0%)	1 (20%)	1
Time to death in days, mean (SD)	143.2 (89.5)	1,394.8 (881.2)	<0.01
Time to death in years, mean (SD)	0.4 (0.3)	3.82 (2.4)	<0.01
Protein count, mean (SD)	1,152 (262)	1,146 (130)	0.90

In the BALF discovery proteomics cohort (*n* = 50), the short survival group mean days to death was 188.3 ( ±109.2) and the long survival group mean days to death was 2,090.8 ( ±974.9) (mean difference of 1,902.5 days, *p* value <0.01). Age, FVC%, and DLCO% were similar across the two groups, although the short survival group had a non-significantly lower proportion of patients on immunosuppression. Most patients in the cohort were male with a history of smoking (Table 2).

**Table 2:** IPF BALF proteomics discovery cohort

	Short survival (*n* = 25)	Long survival (*n* = 25)	*p* value
Age, mean (SD)	66.6 (7.2)	66.8 (6.9)	0.89
Gender, n (%) male	18 (72%)	15 (60%)	0.60
Smoker, n (%) ever	17 (68%)	14 (56%)	0.59
FVC% predicted, mean (SD)	62.6 (11.55)	61.8 (11.34)	0.81
DLCO% predicted, mean (SD)	80.4 (23.70)	88.4 (13.97)	0.18
On immunosuppression, n (%)	6 (24%)	7 (28%)	0.78
Survived to 5 year, %	0 (0%)	18 (72%)	–
Time to death in days, mean (SD)	188.3 (109.2)	2090.8 (974.9)	<0.01
Time to death in years, mean (SD)	0.52 (0.30)	6.0 (2.4)	<0.01
Protein count, mean (SD)	871 (71)	887 (57)	0.70

In the survival confirmation cohort (*n* = 156), the mean age was 66.1 years, and most patients were male (64.1%) ever-smokers (66.7%) (Table 2). The mean (SD) baseline FVC% was 66.3 (17.5) and DLCO% was 50.8 (16.0). Within the 5 years following BALF collection, 69.9% of participants had died. Twenty-nine percent of the cohort was taking immunosuppressing medications, including prednisone, azathioprine, or mycophenolate mofetil, at the time of BALF collection and none were on anti-fibrotic medications (Table 3).

**Table 3:** Confirmation cohorts

	Survival cohort (*n* = 156)	Contemporary cohort (*n* = 52)	*p* value
Age, mean (SD)	66.1 (7.6)	68.7 (5.7)	0.01
Sex, n (% male)	100 (64.1%)	43 (82.7%)	0.02
Smoker, n (% ever)	104 (66.7%)	32 (61.5%)	0.9
FVC% predicted, mean (SD)	66.3 (17.5)	73.2 (16.6)	0.01
DLCO% predicted, mean (SD)	50.8 (15.9)	42.8 (11.9)	<0.01
On immunosuppression, n (%)	45 (28.9%)	0 (0%)	<0.01
On antifibrotic, n (%)	0 (0%)	22 (42.3%)	<0.01
Survived up to 5 year, n %	47 (30.1%)	a9 (17.3%)	0.10
Time to death in days, mean (SD)	1,326.9 (1403.6)	1,269.0 (994.8)	0.8
Time to death in years, mean (SD)	3.6 (3.8)	3.5 (2.7)	0.8
BALF % neutrophil, mean (SD)	8.8 (9.6)	–	–
GAP score, mean (SD)	3.1 (1.1)	4.4 (1.2)	<0.01

SD, standard deviation; FVC, forced vital capacity; DLCO, diffusion capacity for carbon monoxide; BALF, bronchoalveolar lavage fluid; GAP, gender, age, and physiology score. BALF cell differential unavailable for the contemporary cohort.

a Survival data only available for the 50-week period of observation for those patients in the validation dataset whose samples were derived from WRAP-IPF.

IPF patients in the contemporary confirmation cohort (*n* = 52) were older (mean age 68.7 years) and more often male (82.7%) compared to the survival confirmation cohort. Like the survival confirmation cohort, the majority of patients in the contemporary confirmation cohort were ever-smokers (61.5%). Mean baseline FVC% was 73.2 (16.6) and baseline DLCO% was 42.8 (11.9). At the time of BALF collection, 42% of the contemporary confirmation cohort were taking anti-fibrotic medications and none were on immunosuppression (Table 3).

### Lung tissue proteomics discovery pathway analysis

**Figure 1:**
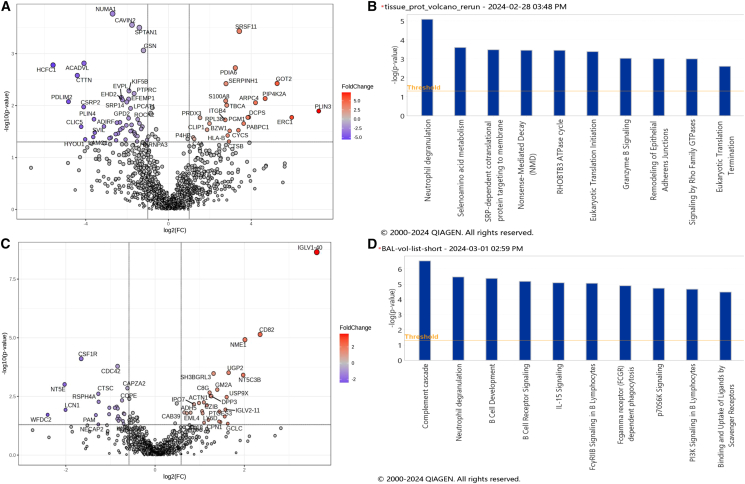
Volcano plots and canonical pathway summary graphs of differentially expressed proteins (DEP) in IPF lung tissue (A) Volcano plot of DEP in lung tissue, |log2(FC)|≥1 (2x FC in either direction); *p* value <0.05. (B) IPA canonical pathway summary graph of tissue DEP showing top 10 pathways by *p* value; *p* value <0.05. (C) Volcano plot of DEP in BALF, |log2(FC)|≥1 (2x FC in either direction); *p* value <0.05. (D) IPA canonical pathway summary graph of BALF DEP showing top 10 pathways by *p* value, *p* value <0.05.

A total of 1,809 proteins were identified in at least one of the 10 tissue samples. Of these, 19 proteins were considered contaminants and excluded from analysis. Differential expression analysis of 1,125 proteins detected in at least 50% of the samples yielded 70 significant proteins exhibiting at least 2-fold change between the short and long survival groups (*p* < 0.05, absolute log2FC ≥ 1) (Figure 1A). Of these 70 proteins, 69 were successfully mapped to ingenuity pathway analysis (IPA) canonical pathways. A total of 95 pathways were determined to be significant (*p* < 0.05), i.e., discriminant proteins between short survival and long survival were found to be significantly enriched in these pathways. The most significant pathway based on *p* value was neutrophil degranulation (*p* value = 8.3E-06, *Z* score = 1.667, number of mapped proteins = 9) (Table S1). The positive *Z* score = 1.667 in this analysis implies an increase in expression of the neutrophil degranulation pathway in the short survival IPF group. Selenoamino acid metabolism was ranked as the second significant pathway (*p* value = 2.5E-04, *Z* score = 0, mapped proteins = 4), and SRP-dependent co-translational protein targeting to membrane was ranked third (*p* value = 3.3E-04, *Z* score = 0, mapped proteins = 4) (Figure 1B, Table S1). The *Z* scores = 0 of these two pathways indicated that IPA could not calculate whether they were up-regulated or down-regulated. The nine proteins belonging to the neutrophil degranulation pathway were CTSB, HLA-B, MVP, PDXK, PGM1, PSMA2, S100A8, SPTAN1, and SRP14 (Table S2).

### BALF discovery proteomics pathway analysis

A total of 1,232 proteins were identified in at least one of the 50 tissue samples. Of these, six proteins were considered contaminants, and 330 proteins were detected in less than 50% of samples and were excluded. Differential expression analysis of 896 non-contaminant proteins found in at least 50 BALF% of samples yielded 83 significant proteins exhibiting at least 1.5-fold change between the short and long survival groups (*p* value <0.05, absolute log2FC ≥ 0.6) (Figure 1C). Of these, 82 molecules were successfully mapped to IPA canonical pathways. A total of 72 pathways were determined to be significant (*p* < 0.05). The top-ranking pathways by *p* value are complement cascade [-log2(*p* value) = 6.552, mapped molecules = 7], followed by neutrophil degranulation [-log2(*p*-value) = 5.466, mapped molecules = 10] (Figure 1D; Table S3). The 10 significantly differentially expressed proteins mapped to neutrophil degranulation were CAB39, CTSC, GM2A, HK3, IST1, JUP, PRG2, SERPINB1, TCN1, and TUBB (Table S4).

### BALF neutrophil degranulation marker levels are associated with worse survival in IPF

The pathway most significantly associated with poor survival in our lung tissue proteomics cohort was neutrophil degranulation and, in our discovery BALF proteomics cohort, neutrophil degranulation was second most significant. Therefore, we elected to carry forward this candidate pathway for additional confirmatory testing using complementary absolute protein quantification methods.

### BALF neutrophil degranulation markers, absolute quantification validation, and exploration of clinical significance

In the survival confirmation cohort, using unadjusted models and four different markers of neutrophil degranulation, higher levels of BALF neutrophil degranulation were associated with worse 5-year survival (DNA-MPO: hazard ratio [HR] = 1.19, 95% confidence interval [CI] 1.05–1.36, *p* < 0.01; exDNA: HR = 1.32, 95% CI 1.15–1.53, *p* < 0.01; calprotectin: HR = 1.27, 95% CI 1.07–1.51, *p* < 0.01; exNE: HR = 1.17, 95% CI 1.04–1.31, *p* = 0.01). In models adjusted for GAP score and use of immunosuppression, DNA-MPO, exDNA, and calprotectin remained significantly associated with worse survival (DNA-MPO: HR = 1.18, 95% CI 1.02–1.38, *p* < 0.05; exDNA: HR = 1.30, 95% CI 1.09–1.55, *p* < 0.01; calprotectin: HR = 1.23, 95% CI 1.00–1.51, *p* < 0.05).

**Figure 2:**
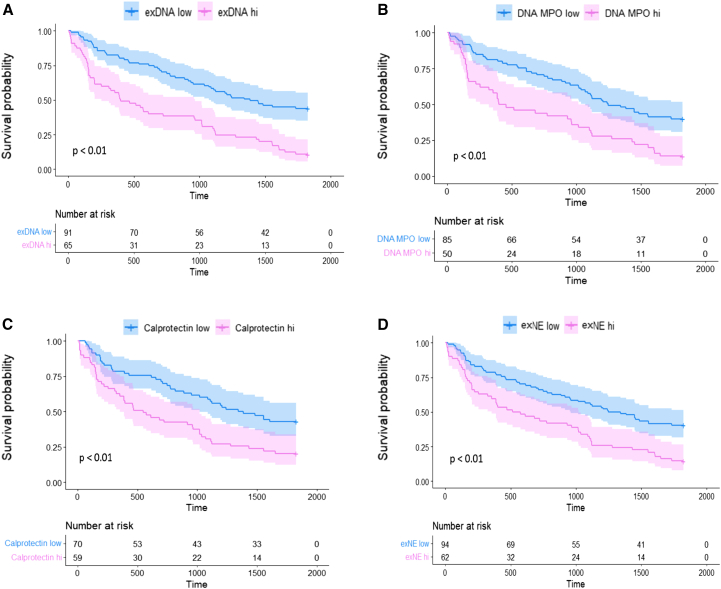
Kaplan-Meier survival curves in survival cohort Each pane represents one of the neutrophil degranulation markers tested within the validation cohort, which was divided into high and low levels by optimal cutoff threshold. Patients with neutrophil degranulation marker levels higher than the optimal cutoff threshold are represented in pink and those with below the threshold in blue. Survival probability is represented by the *y* axis, and the time to death in days is plotted on the *x* axis. (A) shows results from exDNA: HR = 2.57, 95% CI 1.76–3.76, *p* < 0.01; (B) shows results from DNA-MPO: HR = 2.15, 95% CI 1.43–3.24, *p* < 0.01; (C) shows results from calprotectin: HR = 2.00, 95% CI 1.31–3.05, *p* < 0.01; (D) shows results from exNE: HR = 2.06, 95% CI 1.41–3.01, *p* < 0.01. Optimal threshold values for these analyses: (exDNA: 4.32, exDNA median: 4.12), (DNA-MPO: 8.45, DNA-MPO median: 8.28), (calprotectin: 5.43, calprotectin median 5.19), (exNE: 10.90, exNE median: 10.61).

For each neutrophil degranulation marker tested, IPF patients were stratified into two groups based on optimal cutoff thresholds as discussed in the methods section. For all four measures, the group with high levels of BALF neutrophil degranulation markers had significantly worse survival compared to the group with low levels (DNA-MPO: median level 8.28 OD, optimal cutoff threshold 8.45 OD, HR = 2.15, 95% CI 1.43–3.24, *p* < 0.01; exDNA: median level 4.12 ng/mL, optimal cutoff threshold 4.32 ng/mL, HR = 2.57, 95% CI 1.76–3.76, *p* < 0.01; calprotectin: median level 5.19 pg/mL, optimal cutoff threshold 5.43 pg/mL, HR = 2.00, 95% CI 1.31–3.05, *p* < 0.01; exNE: median level 10.61 ng/mL, optimal cutoff threshold 10.90 ng/mL, HR = 2.06, 95% CI 1.41–3.01, *p* < 0.01) (Figure 2). Each of these associations remained significant after adjusting for GAP score and use of immunosuppression (DNA-MPO: HR = 1.98, 95% CI 1.20–3.26, *p* < 0.01; exDNA: HR = 2.19, 95% CI 1.33–3.60, *p* < 0.01; calprotectin: HR = 1.79, 95% CI 1.05–3.03, *p* = 0.03; exNE: HR = 1.82, 95% CI 1.12–2.94, *p* = 0.01).

In the subset with serum available for testing, there was no significant association with worse 5-year survival and serum exDNA (HR = 1.34, 95% CI 0.44–4.10) or serum exNE (HR = 1.09, 95% CI 0.71–1.65) in the survival confirmation cohort.

### BALF neutrophil degranulation marker levels associated with worse lung disease severity in IPF

**Figure 3:**
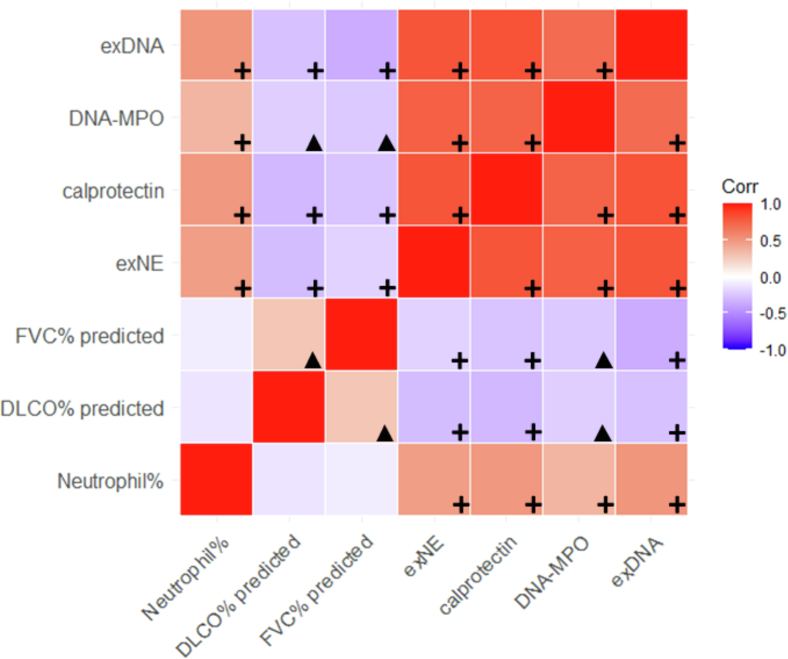
Correlogram highlighting degree of correlation across the four neutrophil degranulation markers, neutrophil percentage in BALF samples, and the negative correlation with FVC and DLCO (% predicted) across the discovery cohort A red box indicates a positive correlation, with darker reds indicating greater strength of correlation, and purple boxes indicate negative correlation, with darker colors indicating stronger correlation, based on Pearson’s correlation coefficient. Black plus signs indicate correlations with *p* value <0.01, and black triangles indicate significant correlations with *p* values between 0.01 and 0.05.

In addition to survival, we evaluated the relationship between BALF neutrophil degranulation markers and disease severity. In the survival confirmation cohort, there was a significant negative correlation between lower FVC% and higher BALF neutrophil degranulation marker levels [MPO-DNA: r=(−)0.19, *p* = 0.03; exDNA: r=(−)0.34, *p* < 0.01; calprotectin: r=(−)0.30, *p* < 0.01; exNE: r=(−)0.22, *p* < 0.01] (Figure 3). Similarly, there was a negative correlation between lower DLCO% and higher BALF neutrophil degranulation marker levels [MPO-DNA: r=(−)0.23, *p* < 0.01; exDNA: r=(−)0.26, *p* < 0.01; calprotectin: r=(−)0.29, *p* < 0.01; exNE: r=(−)0.22, *p* < 0.01] (Figure 2). Using linear regression models adjusted for age and sex, each neutrophil degranulation marker remained significantly negatively associated with FVC% (MPO-DNA: *p* = 0.02; exDNA: *p* < 0.01; calprotectin: *p* < 0.01; exNE: *p* < 0.01) and DLCO% (MPO-DNA: *p* = 0.01; exDNA: *p* < 0.01; calprotectin: *p* < 0.01; exNE: *p* = 0.01).

In the subset with serum tested, there was no significant correlation between FVC% and serum exDNA (*p* = 0.79) or exNE (*p* = 0.21) as well as no correlation between DLCO% and serum exDNA (*p* = 0.17) or exNE (*p* = 0.66).

### Correlation of BALF neutrophils and neutrophil degranulation markers

In the survival confirmation cohort, the levels of each BALF neutrophil degranulation marker significantly correlated with the percentage of BALF neutrophils (DNA-MPO: r = 0.34, *p* < 0.01; exDNA: r = 0.49, *p* < 0.01; calprotectin: r = 0.51, *p* < 0.01; exNE: r = 0.46, *p* < 0.01) (Figure 3). There was also a strong positive correlation among the four different measures of BALF neutrophil degranulation (Figure 3).

### Contemporary confirmation cohort

In the contemporary confirmation cohort, there was a significant relationship between higher BALF neutrophil degranulation levels (quantified by exDNA) and lower FVC% [r=(−)0.29, *p* = 0.04] and lower DLCO% [r=(−)0.30, *p* = 0.04].

## Discussion

This study identified neutrophil degranulation as a key pathway associated with IPF survival. Using untargeted quantitative proteomics in IPF lung and microenvironment and confirming with absolute quantification in BALF from a large cohort, we demonstrated a strong relationship between elevated neutrophil degranulation and poor survival. Cross-sectional data from two IPF cohorts also showed associations between higher BALF neutrophil degranulation markers and worse baseline disease severity, supporting the clinical relevance of these findings in contemporary settings. Our findings in BALF are of particular interest, as it is a more accessible compartment (BALF vs. lung tissue) that can allow for future neutrophil-focused research in IPF patients.

**Figure 4:**
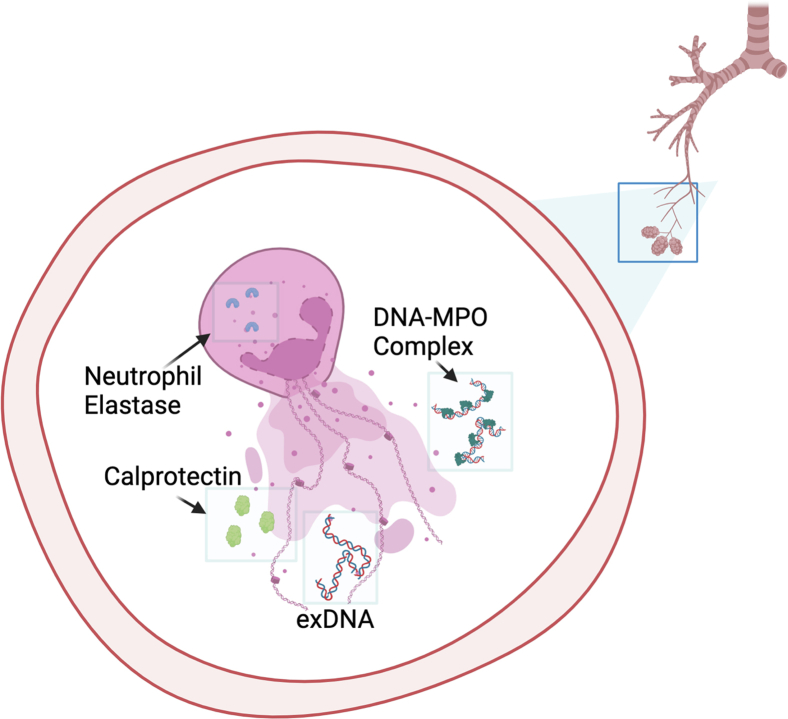
Neutrophils in the lungs after undergoing stimulation to release their intracellular protein cargo via degranulation This material includes extracellular DNA (exDNA), calprotectin, and DNA-myeloperoxidase (DNA-MPO) complexes. Neutrophil degranulation is mediated by the activity of neutrophil elastase, which is also detectable in the extracellular environment. The presence of exDNA has been associated with immunologic and inflammatory reactions that likely result in direct lung and airway injury.

Previous studies highlighted the prognostic role of BALF neutrophilia and serum neutrophil-to-lymphocyte ratios in IPF patients.^11^^,^^12^ However, the mechanism by which the presence of neutrophils results in worse outcomes in IPF is unknown. Our data suggest neutrophil activation via degranulation as the possible link. A notable subtype of neutrophil degranulation is NET formation (NETosis), which can be measured by DNA-MPO protein complexes and calprotectin where activated neutrophils expel their DNA and cytoplasmic proteins such as NE extracellularly (Figure 4). These NET scaffolds play a direct role in innate immune functions to clear pathogenic debris but have also been implicated in lung injury and associated with worse prognosis in asthma, bronchiectasis, and acute lung injury.^13^^,^^14^^,^^15^ NETs also induce key pathways of pulmonary fibrosis,^16^^,^^17^^,^^18^^,^^19^ and NET inhibition in animal models can resolve lung injury and fibrosis,^16^^,^^20^ and this study provides in-human evidence that neutrophil degranulation, including NETosis, may contribute to IPF prognosis.

There are several links between neutrophil degranulation and known IPF risk factors. For instance, smoking and genetic variants associated with increased MUC5B production, which are known risk factors for IPF, can induce neutrophil degranulation and NETosis.^21^^,^^22^^,^^23^^,^^24^^,^^25^ Additionally, NET-derived proteins such as histones and myeloperoxidase (MPO) have been found to be cytotoxic to alveolar epithelial cells,^19^ and NETs have been shown to induce lung fibroblast proliferation and differentiation,^16^^,^^17^ further supporting the role of neutrophil-derived NETs in lung injury and fibrosis in IPF.

Our findings also offer a possible explanation for the increased autoantibodies observed in IPF patients.^26^^,^^27^^,^^28^^,^^29^ Neutrophil degranulation and NETosis are associated with autoantigen exposure and autoantibody development in diseases like rheumatoid arthritis and scleroderma.^30^^,^^31^^,^^32^^,^^33^^,^^34^^,^^35^ This suggests that neutrophil activity may be a key factor in the complex relationship between IPF and autoimmune interstitial lung diseases (ILDs).^36^^,^^37^^,^^38^^,^^39^^,^^40^

In a subset of patients from the survival confirmation cohort with matched serum samples, we evaluated circulating neutrophil degranulation markers (exDNA and exNE) to explore whether systemic neutrophil activity correlates with disease severity or survival in IPF. Interestingly, these circulating markers were *not associated with FVC%, DLCO%, or survival*, in contrast to their BALF counterparts. These findings suggest that neutrophil activation in IPF may be localized to the lung microenvironment rather than systemically disseminated. While these analyses were limited by a relatively small sample size (*n* = 57), they raise the intriguing possibility that lung-based assessments may capture compartment-specific pathobiology that is not reflected in blood-based markers. This reinforces the utility of BALF in mechanistic IPF studies, even as challenges remain for broader clinical implementation.

### Limitations of the study

There are important considerations to interpreting these data that require future prospective study. For instance, samples included in the survival cohort were collected in a previous era of IPF treatment (immunosuppression); for these reasons, we included a second, multi-center cohort to confirm the relationship between neutrophil degranulation markers and disease severity under contemporary treatment practices. Additionally, because BALF is rarely obtained for clinical or research purposes in IPF in current practice, it may be challenging to incorporate these findings into clinical practice despite the reported safety in IPF patients.^41^

While these data show a strong association between neutrophil degranulation and IPF outcomes, it remains unclear whether this process is directly pathogenic or a marker of upstream lung injury. Additionally, it is unknown whether the increased neutrophil degranulation in some patients is due to more prone neutrophils or greater exposure to degranulation-inducing factors. Future studies should explore these functional questions via direct visualization of neutrophil degranulation under various conditions in lung tissue and BALF as well as proteomic and transcriptomic analyses of isolated neutrophils from individuals with IPF. Prospective studies with uniform sample collection will be needed to assess if neutrophil degranulation or NETosis can serve as a biomarker for prognosis and potentially guide treatment decisions in IPF. Future studies should also investigate circulating neutrophil activation markers, as bronchoscopy is not routinely performed in clinical practice.

In conclusion, neutrophil degranulation is an underexplored mechanism of lung injury in IPF. Our data suggest that it may contribute to worse outcomes, and future research should determine if it represents a novel therapeutic target or a biomarker for disease progression in IPF.

## Resource availability

### Lead contact

Requests for further information and resources should be directed to and will be fulfilled by the lead contact, Scott M. Matson (smatson@kumc.edu).

### Materials availability

This study did not generate new unique reagents.

### Data and code availability

• Raw proteomics data have been deposited at MassIVE: MSV00098032 and ProteomeXchange: PXD064413.
• This paper does not report original code.
• Additional resources: BALF samples are from WRAP-IPF Clinical trial registry number: NCT01982968.
• Any additional information required to reanalyze the data reported in this paper is available from the lead contact upon request.

## Acknowledgments

We would like to acknowledge Hal Collard and the rest of the Weighing Risks and Benefits of Laparoscopic Anti-Reflux Surgery in Patients with IPF (WRAP-IPF) investigators for contributing samples for our validation cohort. We would also like to acknowledge Kevin K. Brown for his contribution and efforts to collect and maintain the original NJH cohort. This work was funded by P20 GM130423 (S.M.M.), R01 AR076450, and Pfizer ASPIRE Investigator Initiated Grant (WI227190) (M.K.D.).

## Author contributions

S.M.M., J.J.S., and M.K.D. contributed to the study conception and design. M.K.D., P.J.W., and J.J.S. contributed to the recruitment of the study subjects. S.M.M., J.S.L., P.J.W., M.K.D., J.J.S., L.T.N., Y.S., V.F., C.L., I.A., M.P.W., M.J.R., and M.K.D. contributed to acquisition of the data. S.M.M., L.T.N., A.A., E.N., D.K., and M.K.D. contributed to the analysis and interpretation of the data. S.M.M. and M.K.D. contributed to the initial drafting of the manuscript. All authors contributed to critical revision and final approval of the manuscript.

## Declaration of interests

J.J.S. has investigator-initiated grants from Boehringer Ingelheim and Pfizer. He has received consulting fees for trial design from Istesso and 3+2 Pharma. He has received payment/honoraria for disease state education from Boehringer Ingelheim. He has received payment for expert testimony from Cooper Rice and Olson and Childs McCune. He has participated in a Data Safety Monitoring Board at the University of Pittsburgh. J.L. received grants from Boehringer Ingelheim. She has received consulting fees from Blade, Avalyn, Boehringer Ingelheim, United Therapeutics, and Eleven P15. She has participated on a Data Safety Monitoring Board or Advisory Board for United Therapeutics and Avalyn Pharma. She is in a leadership role at the Pulmonary Fibrosis Foundation. She has received a research gift from Pliant Therapeutics. M.K.D. has received an investigator-initiated grant from Pfizer. She has received grants from Pfizer and Boehringer Ingelheim. P.J.W. has received grants from Boehringer Ingelheim, the NIH, Roche, Sanofi, and Pliant. He has received consulting fees from Blade Therapeutics. He has received honoraria from Boehringer Ingelheim. The proteomics data were collected in the Mass Spectrometry and Proteomics Core facility utilizing the Orbitrap Ascend Tribrid System that was purchased with funds provided by the University of Kansas Cancer Center, which is supported by the National Cancer Institute Cancer Center Support Grant P30 CA168524.

## STAR★Methods

### Key resources table

**Table:** 

REAGENT or RESOURCE	SOURCE	IDENTIFIER
**Antibodies**		

anti-human MPO antibody	Bio-Rad Antibodies	Bio-Rad0400-0002; RRID: AB_10152916

**Biological samples**		

IPF Lung Tissue	National Jewish Lung Biorepository	https://www.nationaljewish.org/research-science/research-support/biobanks/integrated-bioinformation-specimen-center
IPF BALF Samples	National Jewish Lung Biorepository	https://www.nationaljewish.org/research-science/research-support/biobanks/integrated-bioinformation-specimen-center
WRAP-IPF BALF Samples	Raghu et al.^3^	Clinical trial registry number: NCT01982968
UCSF IPF biorepository BALF Samples	UCSF Lung Tissue and Lung Sample Biorepository	https://profiles.ucsf.edu/paul.wolters

**Chemicals, peptides, and recombinant proteins**		

3,3′,5,5′-TMB substrate	Invitrogen	Cat Number: 34021
2N sulfuric acid stop solution	Sigma Aldrich	CAS Number: 7664-93-9
1% BSA in PBS	N/A	N/A

**Critical commercial assays**		

dsDNA assay	Invitrogen Quanti-iT^TM^ PicoGreen®	https://www.thermofisher.com/order/catalog/product/P7589
Calprotectin	Werfen	https://www.werfen.com/na/en/quanta-flash-calprotectin-reagents
exNE	Abcam	https://www.abcam.com/en-us/products/elisa-kits/human-neutrophil-elastase-elisa-kit-ab270204
Cell death detection kit	Abcam	https://www.abcam.com/en-us/products/assay-kits/live-and-dead-cell-assay-ab115347

**Deposited data**		

Tissue and Lung Proteomics Data	MassIVE	MSV00098032 https://massive.ucsd.edu/ProteoSAFe/dataset.jsp?task=6213daf718b64f10bedf38105d7a0eec https://doi.org/10.25345/C5GM8219Q
Tissue and Lung Proteomics Data	ProteomeXchange	PXD064413

**Software and algorithms**		

Surv_cutpoint( ) function	Analysis method	https://search.r-project.org/CRAN/refmans/survminer/html/surv_cutpoint.html
Qiagen IPA pathway analysis	Pathway model	https://digitalinsights.qiagen.com/products-overview/discovery-insights-portfolio/analysis-and-visualization/qiagen-ipa/?gad_source=1&gad_campaignid=21524076944&gbraid=0AAAAADbyWl1XU0Rboj6d1ghBmIkYq9DGc&gclid=Cj0KCQjw_L_FBhDmARIsAItqgt7s7kRh0bQUJVyitUeEqe4QhW5UNvv20XsowdXJBEIDT_9eG56cv7saArNuEALw_wcB

### Experimental model and study participant details

This study was approved by the Institutional Review Board (IRB) of University of Kansas Medical Center (STUDY00146636) on 4/16/2021. Informed consent was obtained at the time of sample collection for inclusion in the interstitial lung disease biorepository at National Jewish Health (NJH).

#### Lung tissue and BALF proteomics discovery cohort

We obtained surgical lung biopsy tissue samples from the interstitial lung disease biorepository at NJH that included patients who were prospectively enrolled into the NJH Center of Research Study. A subset of these patients have been previously reported on.^11^ We included 50 patients from this biorepository who had undergone BALF collection and 10 patients who had undergone prospective research lung biopsy, who met the current IPF diagnostic criteria,^42^ which was confirmed by contemporary chart review, and who had a date of death confirmed using the United States Center for Disease Control’s National Death Index (CDC NDI). All samples were collected between 1986 and 2009. Clinical and demographic information was extracted from the medical record to include demographics, medications, and pulmonary function testing (PFT) results at the time of tissue collection ( ±90 days). To best distinguish features associated with poor survival, a ‘short survival’ group and a ‘long survival’ group were matched based on baseline features (age and baseline disease severity as determined by percent predicted forced vital capacity (FVC%) at time of procedures) (Table 1). All tissue and BALF samples underwent label-free quantitative proteomics, as described below.

#### Survival confirmation cohort

To confirm pathways identified in the tissue proteomics analysis that were associated with IPF survival, we included all the remaining IPF patients from the NJH biorepository with adequate BALF volumes available (*N* = 156). This cohort was chosen for survival confirmation because the time frame of sample collection (1986–2009) allowed for nearly complete survival data for each participant and permitted a robust survival analysis of any candidate target. As described above, all subjects met current IPF diagnostic criteria (confirmed by contemporary chart review), clinical and demographic information was extracted from the medical record, and date of death was confirmed using the CDC NDI.

#### Contemporary confirmation cohort

Given the era of collection of the survival confirmation cohort, a portion of IPF patients were on immunomodulatory therapy (29%) and none were on anti-fibrotic medications, which differs from the current clinical approaches to IPF management. To confirm contemporary applicability of any pathways of interest identified in the proteomics analysis, we included a contemporary IPF BALF confirmation cohort. This cohort included 27 IPF BALF samples from the University of California San Francisco (UCSF) ILD biorepository and 25 IPF BALF samples from the Weighing Risks and Benefits of Laparoscopic Anti-Reflux Surgery in Patients with IPF (WRAP-IPF) cohort.^43^ UCSF samples were collected between 2013 and 2018 and WRAP-IPF samples were collected between 2014 and 2016. Clinical and PFT data were collected as part of the research study. Survival data was insufficient in this cohort, therefore, analyses with this cohort were limited to cross-sectional relationships with disease severity but provide an important assessment of relevance to contemporary IPF patients.

#### Lung tissue collection

During the initial prospective enrollment of the Specialized Center of Research Study at NJH, each patient underwent research protocol open thoracotomy or video-assisted thoracoscopic lung biopsy to confirm the usual interstitial pneumonia pattern. These biopsies were taken from at least two separate sites in the same lung (upper and lower lobes) as previously reported.^11^

#### BALF collection

BALF was collected using saline lavage. Cell count and differential were determined using an unspun BALF aliquot in the survival confirmation cohort. BALF cell-free supernatant was obtained following centrifugation and stored at −80°C until analysis.

*Pulmonary function testing* was performed under normal conditions in routine clinical care, for prospective research purposes, or in the case of WRAP-IPF subjects, prior to surgical procedure. FVC% and percent predicted diffusing capacity for carbon monoxide (DLCO%) at the time of BALF collection ( ±3 months) were used to determine baseline disease severity.

### Method details

#### Lung tissue protein isolation

Fresh frozen lung tissues were sliced, weighed, and thawed on ice. RIPA buffer spiked with protease inhibitors and phosphatase inhibitors was added to tissue samples (30 μL RIPA/1 mg tissue). The samples were then homogenized using BeatBox homogenizer (PreOmics) and manufacturer’s kit on standard power for 10 min twice. Tissue lysates were centrifuged at 14,000g for 10 min and supernatants free of cellular remnants and particulates were transferred to fresh tubes for protein isolation.

Total protein level for each tissue lysate was quantified using the Pierce BCA protein assay following manufacturer’s protocol against a standard curve of BSA. Standards and samples were measured with a plate reader at an absorbance of 562 nm. Proteins from tissue lysates were then isolated using acetone precipitation. An adequate volume of supernatant containing at least 50 μg protein was aliquoted, reduced, alkylated, and proteins then precipitated with 3x volume ice-cold acetone overnight at −20^o^C. The protein was pelleted and digested overnight with trypsin.

#### BALF protein isolation

BALF samples were thawed, vortexed and centrifuged at 5000 x *g* for 5 min. A 100 μL aliquot of supernatant was transferred to a fresh microcentrifuge tube for protein isolation. Samples were reduced with the addition of 0.5 M TCEP to a final concentration of 5 mM followed by incubation at 37 °C for 30 min. Reduced samples were alkylated with the addition of 375 mM iodoacetamide to a final concentration of 10 mM followed by incubation in the dark at room temperature for 30 min. Ice-cold acetonitrile (ACN) was added to each sample to a volume ratio of 3:1. Samples were vortexed and stored at −20 °C overnight. After precipitation, samples were centrifuged at 14,000 x *g* at 4 °C for 30 min to pellet the proteins. The supernatant was removed, and the pellet was air dried on benchtop for 10 min. The proteins were resuspended in 50 mM TEAB pH 8. Trypsin was added (500 ng) and the proteins were allowed to digest overnight at 37 °C with shaking at 500 RPM (Thermomixer, Eppendorf). The digestion was quenched with the addition of 10% formic acid to a final concentration of 1%. Digested samples were centrifuged at 10,000 x *g* for 10 min to remove particulates and the supernatant was transferred to a fresh tube and stored at −20 °C until mass spectrometry analysis. Peptide concentration was measured using a Nanodrop spectrophotometer (Thermo Scientific) at 205 nm prior to LC-MS/MS analysis.

#### Label-free quantitative proteomics

Digested peptides from lung tissue and BALF were analyzed by nanoLC-MS/MS using a Vanquish *Neo* nano-UPLC interfaced directly to the Orbitrap Ascend Tribrid mass spectrometer (Thermo Fisher) equipped with a FAIMS source (details in Supplement Methods). Spectra were searched with Proteome Discoverer 3.0 against the human database downloaded from Uniprot on May 5, 2023, and a database of 155 common contaminants.

#### Neutrophil degranulation activity marker testing

All BALF cell-free supernatant from the survival confirmation cohort was tested for levels of neutrophil degranulation using four complementary, but distinct, absolute quantitative methods.^14^^,^^34^^,^^35^^,^^44^^,^^45^^,^^46^^,^^47^^,^^48^^,^^49^^,^^50^^,^^51^ These assays included a research-based sandwich ELISA for neutrophil extracellular traps (NET) proteins [DNA-myeloperoxidase (MPO)], an immunofluorescence assay for extracellular DNA (exDNA; Invitrogen Quanti-iT PicoGreen dsDNA assay), an ELISA for calprotectin (Werfen), and an ELISA for extracellular NE (exNE; Abcam). Given limitations of BALF volume in the contemporary confirmation cohort, only BALF exDNA was tested in this cohort as it required the smallest amount of BALF. A subset of participants from the survival confirmation cohort had serum available (*N* = 57), which were tested for exDNA and exNE. For the commercially available assays, exDNA, calprotectin, and exNE, manufacturers protocols for testing were followed. For DNA-MPO, previously published protocols were used^31^ where a high-binding EIA/RIA 96-well plate (Costar) was coated overnight at 4°C with anti-human MPO antibody (Bio-Rad0400-0002) in coating buffer from Cell Death Detection ELISA kit (Roche). The plate was then washed three times with 0.05% Tween 20 in PBS and blocked with 1% BSA in PBS for 1 h at room temperature (RT). Following three more washes, BALF samples were diluted 1:2 in the blocking buffer and incubated for 1 h at RT. The plate was then washed five times, followed by incubation with anti-DNA antibody for 1 h at RT (HRP-conjugated, Cell Death kit, diluted 1:100 in blocking buffer). After 5 more washes, the plate was developed with 3,3′,5,5′-TMB substrate (Invitrogen) followed by a 2N sulfuric acid stop solution. Absorbance was measured at a wavelength of 450 nm. A background control well was run for each sample that included the BALF sample without including the primary anti-MPO antibody and background absorbance level was subtracted from each sample to account for any non-specific background binding of the sample.

### Quantification and statistical analysis

#### Proteomics data analysis

To identify discriminating lung tissue and BALF proteins between the short survival versus long survival groups, contaminant proteins and proteins detected in less than 50% of samples were excluded from analysis. Protein abundance data was normalized, log2 transformed, and median centered. For each protein, fold change (FC) and *p*-value between short survival and long survival was calculated. Proteins considered differentially expressed must have a *p*-value ≤0.05 and an absolute log_2_FC ≥ 1 (at least 2-fold change in either direction). Differentially expressed proteins were utilized to perform an overrepresentation analysis (ORA) using QIAGEN Ingenuity Pathway Analysis (IPA).^52^

The Core Analysis workflow was used to generate the Canonical Pathways results. Pathways were ranked based on *p*-value, and a pathway with an overlap *p*-value ≤0.05 was considered statistically significant. IPA also considers expression changes to predict pathway activation: A positive *Z* score suggests activation and a negative *Z* score suggests inhibition; however, a *Z* score = 0 or NaN (not a number) does not necessarily imply a prediction of no alternation. All z-scores were included in analysis per IPA recommendation (Tables S1 and S2).

#### Survival analysis

In the survival confirmation cohort, Cox proportional-hazards (PH) models were used to assess the association between survival time (up to 5-year) and log transformed BALF DNA-MPO, exDNA, calprotectin, and exNE levels. Multivariate Cox PH models were used to determine associations with death adjusting for GAP score,^53^ use of immunosuppressing medications (i.e., corticosteroids, mycophenolate mofetil, azathioprine, cyclophosphamide). We also dichotomized the survival confirmation cohort into high and low BALF neutrophil degranulation marker groups using a cut point function that determines the optimal point for discriminating between two groups for a continuous variable using the maximal log-rank statistic using the surv_cutpoint() function in R, with a minimal proportion of observations per group set at 0.15. In this cut point analysis, we determined optimal levels for discriminating survival associated with each BALF neutrophil degranulation marker. Any patient with a BALF neutrophil degranulation marker level greater than the optimal cut point was categorized into the high group, and those with a level less than the cut point were categorized into the low group. Kaplan-Meier curves were used to visualize the survival function for high and low neutrophil degranulation marker groups. In all models, a *p*-value <0.05 was considered statistically significant.

#### Confirmation cohorts

Correlations between log transformed levels of neutrophil degranulation markers (DNA-MPO, exDNA, calprotectin, and exNE), BALF% neutrophils, FVC% and DLCO% were calculated using Pearson’s correlation coefficient. Linear regression models were used to compare FVC% and DLCO% with log transformed BALF DNA-MPO, exDNA, calprotectin, and exNE levels, sex, and age.

All statistical analysis were performed in R version 4.1.3 unless specified otherwise.
