# Supplementary material for: Neutrophil degranulation in the lung microenvironment linked to idiopathic pulmonary fibrosis severity and survival
Source: iScience. 2025 Nov 19;29(1):114125. doi: 10.1016/j.isci.2025.114125 (PMC12794420; doi:10.1016/j.isci.2025.114125)
Supplement: Document S1. Tables S1–S4 [file mmc1.pdf]

iScience, Volume *29*

Supplemental information

Neutrophil degranulation in the lung microenvironment linked to idiopathic pulmonary fibrosis severity and survival

Scott M. Matson, Linh T. Ngo, Yui Sugawara, Veani Fernando, Claudia Lugo, Angela Kaczorowski-Worthley, Imaan Azeem, Alexis Harrison, Alex Alsup, Emily Schueddig, Devin Koestler, Michaella J. Rekowski, Paul J. Wolters, Joyce S. Lee, Michael P. Washburn, Joshua J. Solomon, and M. Kristen Demoruelle

Supplement table 1. IPA pathway analysis summary of differentially expressed proteins in IPF lung tissue
© 2000-2024 QIAGEN. All rights reserved.

Ingenuity (pval		-log(p-valı	Ratio	z-score	Molecules
Neutrophil	8.317638E-06	5.08	0.0189	1.667	CTSB,HLA-B,MVP,PDXK,PGM1,PS
Selenoami	2.511886E-04	3.6	0.0374	0	HNMT,RPL12,RPL38,RPS3A
SRP-depen	3.311311E-04	3.48	0.0348	0	RPL12,RPL38,RPS3A,SRP14
Nonsense-	3.548134E-04	3.45	0.0342	1	PABPC1,RPL12,RPL38,RPS3A
RHOBTB3 /	3.548134E-04	3.45	0.2	#NUM!	CUL3,PLIN3
Eukaryotic	4.168694E-04	3.38	0.0328	1	PABPC1,RPL12,RPL38,RPS3A
Granzyme	9.332543E-04	3.03	0.125	#NUM!	CYCS,NUMA1
Remodelin	9.772372E-04	3.01	0.0441	#NUM!	ARPC4,CLIP1,NME1
Signaling b	1.000000E-03	3	0.0187	#NUM!	ARPC4,CLIP1,ITGB4,PIP4K2A,SEPT
Eukaryotic	2.454709E-03	2.61	0.0319	#NUM!	RPL12,RPL38,RPS3A
Eukaryotic	2.570396E-03	2.59	0.0316	#NUM!	RPL12,RPL38,RPS3A
Clathrin-m	2.951209E-03	2.53	0.0192	#NUM!	ARPC4,CTTN,ITGB4,S100A8
Response (	3.235937E-03	2.49	0.0291	#NUM!	RPL12,RPL38,RPS3A
Triglycerid	4.265795E-03	2.37	0.0588	#NUM!	GPD2,PLIN3
EIF2 Signal	4.265795E-03	2.37	0.0174	#NUM!	PABPC1,RPL12,RPL38,RPS3A
Class I MH(	4.365158E-03	2.36	0.0133	1.342	CUL3,HECTD1,HLA-B,PSMA2,S10(
Regulation	4.365158E-03	2.36	0.0261	#NUM!	ARPC4,ITGB4,PIP4K2A
Signaling b	4.466836E-03	2.35	0.0259	#NUM!	ENAH,PRKAR2A,PSMA2
Detoxificat	5.011872E-03	2.3	0.0541	#NUM!	CYCS,PRDX3
Gene and (	5.011872E-03	2.3	0.0541	#NUM!	CA1,TALDO1
LXR/RXR A	5.248075E-03	2.28	0.0244	#NUM!	C4A/C4B,S100A8,SAA1
RHOA Sign	5.370318E-03	2.27	0.0242	#NUM!	ARPC4,PIP4K2A,SEPTIN9
Glycerol-3-	5.754399E-03	2.24	0.5	#NUM!	GPD2
ROBO SLIT	5.754399E-03	2.24	0.0236	#NUM!	ARPC4,ENAH,PRKAR2A
RAC Signal	7.079458E-03	2.15	0.0219	#NUM!	ARPC4,ITGB4,PIP4K2A
XBP1(S) ac	8.317638E-03	2.08	0.0417	#NUM!	ACADVL,PDIA6
Glutamate	8.511380E-03	2.07	0.333	#NUM!	GOT2
Aspartate I	8.511380E-03	2.07	0.333	#NUM!	GOT2
MYC Medi	8.912509E-03	2.05	0.04	#NUM!	CYCS,PRKAR2A
Sensory pr	1.071519E-02	1.97	0.0364	#NUM!	CLIC5,SPTAN1
CSDE1 Sigr	1.122018E-02	1.95	0.0357	#NUM!	CTTN,PABPC1
L-cysteine	1.148154E-02	1.94	0.25	#NUM!	GOT2
Assembly (	1.318257E-02	1.88	0.0328	#NUM!	CTSB,ITGB4
Netrin Sigr	1.348963E-02	1.87	0.0173	#NUM!	ARPC4,ENAH,PRKAR2A
UFMylatio	1.348963E-02	1.87	0.0323	#NUM!	DDRGK1,PABPC1
Mitochonc	1.445440E-02	1.84	0.0312	#NUM!	CYCS,HCFC1
Tight Junct	1.479108E-02	1.83	0.0167	#NUM!	CGN,PRKAR2A,SPTAN1
Regulation	1.584893E-02	1.8	0.0163	#NUM!	ITGB4,PABPC1,RPS3A
Collagen b	1.584893E-02	1.8	0.0299	#NUM!	PPIB,SERPINH1
Major patł	1.621810E-02	1.79	0.0161	#NUM!	RPL12,RPL38,RPS3A
Sensory pr	1.659587E-02	1.78	0.029	#NUM!	CLIC5,SPTAN1
Pentose Pł	1.698244E-02	1.77	0.167	#NUM!	TALDO1
Glycerol D	1.698244E-02	1.77	0.167	#NUM!	GPD2
PPARα/RXI	1.819701E-02	1.74	0.0155	#NUM!	GOT2,GPD2,PRKAR2A
Caveolar-n	1.949845E-02	1.71	0.0267	#NUM!	HLA-B,ITGB4

Erythrocyt	1.995262E-02	1.7	0.143	#NUM!	CA1
Pentose pl	1.995262E-02	1.7	0.143	#NUM!	TALDO1
Aspartate	1.995262E-02	1.7	0.143	#NUM!	GOT2
Histidine c	2.238721E-02	1.65	0.125	#NUM!	HNMT
NFE2L2 reg	2.238721E-02	1.65	0.125	#NUM!	TALDO1
Integrin Sig	2.290868E-02	1.64	0.0142	#NUM!	ARPC4,CTTN,ITGB4
BAG2 Signa	2.454709E-02	1.61	0.0235	#NUM!	CTSB,PSMA2
RHOGDI Si	2.511886E-02	1.6	0.0136	#NUM!	ARPC4,ITGB4,PIP4K2A
Hedgehog	2.511886E-02	1.6	0.0233	#NUM!	CUL3,PSMA2
Lipophagy	2.511886E-02	1.6	0.111	#NUM!	PLIN3
KEAP1-NFE	2.818383E-02	1.55	0.022	#NUM!	CUL3,PSMA2
Factors inv	2.818383E-02	1.55	0.022	#NUM!	EHD2,PRKAR2A
ABRA Signa	2.884032E-02	1.54	0.0217	#NUM!	CALD1,SMTN
Actin Nucle	2.884032E-02	1.54	0.0215	#NUM!	ARPC4,ITGB4
Death Rece	3.090295E-02	1.51	0.0208	#NUM!	CYCS,SPTAN1
Erythrocyt	3.090295E-02	1.51	0.0909	#NUM!	CA1
RHO GTPas	3.090295E-02	1.51	0.0909	#NUM!	KIF5B
Aspartate a	3.090295E-02	1.51	0.0909	#NUM!	GOT2
Pentose Pl	3.090295E-02	1.51	0.0909	#NUM!	TALDO1
Glycogen D	3.090295E-02	1.51	0.0909	#NUM!	PGM1
GDP-gluco	3.090295E-02	1.51	0.0909	#NUM!	PGM1
Reversible	3.388442E-02	1.47	0.0833	#NUM!	CA1
Glucose ar	3.388442E-02	1.47	0.0833	#NUM!	PGM1
COPI-medi	3.467369E-02	1.46	0.0196	#NUM!	SPTAN1,USO1
Integratior	3.467369E-02	1.46	0.0194	#NUM!	PRKAR2A,TALDO1
Mitotic Prc	3.467369E-02	1.46	0.0194	#NUM!	NUMA1,USO1
Apoptosis	3.548134E-02	1.45	0.0192	#NUM!	CYCS,SPTAN1
Trafficking	3.630781E-02	1.44	0.0769	#NUM!	CTSB
Advanced	3.630781E-02	1.44	0.0769	#NUM!	SAA1
Glycogen D	3.630781E-02	1.44	0.0769	#NUM!	PGM1
Extracellul	3.715352E-02	1.43	0.0189	#NUM!	ITGB4,NID2
Post-transl	3.801894E-02	1.42	0.0187	#NUM!	C4A/C4B,PDIA6
RAF/MAP I	3.890451E-02	1.41	0.0115	#NUM!	CUL3,PSMA2,SPTAN1
Glutamate	3.890451E-02	1.41	0.0714	#NUM!	GOT2
Role of IL-1	3.890451E-02	1.41	0.0714	#NUM!	S100A8
Phenylalar	3.890451E-02	1.41	0.0714	#NUM!	GOT2
RHO GTPas	3.981072E-02	1.4	0.00889	-1	CUL3,DDRGK1,HINT2,SPTAN1
Hedgehog	4.168694E-02	1.38	0.0177	#NUM!	PRKAR2A,PSMA2
Protein Ub	4.365158E-02	1.36	0.011	#NUM!	HLA-B,PSMA2,USO1
Insulin Sec	4.365158E-02	1.36	0.0109	#NUM!	PABPC1,PRKAR2A,SRP14
Glutaryl-Cc	4.466836E-02	1.35	0.0625	#NUM!	CA1
Parkinson's	4.466836E-02	1.35	0.0625	#NUM!	CYCS
Virus Entry	4.466836E-02	1.35	0.0169	#NUM!	HLA-B,ITGB4
Signaling b	4.786301E-02	1.32	0.0588	#NUM!	NELFB
Neuroprot	4.786301E-02	1.32	0.0164	#NUM!	HLA-B,PRKAR2A
Processing	4.897788E-02	1.31	0.0105	#NUM!	HNRNPA3,HNRNPM,SRSF11
Regulation	4.897788E-02	1.31	0.0161	#NUM!	C4A/C4B,PDIA6
TCR signali	5.011872E-02	1.3	0.0159	#NUM!	ENAH,PSMA2

MHC class	5.011872E-02	1.3	0.0159	#NUM!	CTSB,KIF5B
Clathrin-m	5.370318E-02	1.27	0.0154	#NUM!	ARPC4,CTTN
Inflammas	5.623413E-02	1.25	0.05	#NUM!	CTSB
Role of PKI	5.754399E-02	1.24	0.0147	#NUM!	CYCS,NPM1
RHO GTPa	5.888437E-02	1.23	0.0476	#NUM!	CTTN
Regulation	5.888437E-02	1.23	0.0476	#NUM!	S100A8
Histamine	5.888437E-02	1.23	0.0476	#NUM!	HNMT
DHCR24 Si	5.888437E-02	1.23	0.0146	#NUM!	C4A/C4B,SAA1
GABAergic	6.025596E-02	1.22	0.0144	#NUM!	GOT2,PRKAR2A
Interconve	6.165950E-02	1.21	0.0455	#NUM!	NME1
RHO GTPa	6.165950E-02	1.21	0.0455	#NUM!	S100A8
Chaperone	6.165950E-02	1.21	0.0455	#NUM!	PLIN3
Nephrin fa	6.309573E-02	1.2	0.0435	#NUM!	SPTAN1
Pyrimidine	6.309573E-02	1.2	0.0435	#NUM!	NME1
Tryptophan	6.309573E-02	1.2	0.0435	#NUM!	CA1
Sulfur amir	6.606934E-02	1.18	0.0417	#NUM!	GOT2
Tumoricida	6.606934E-02	1.18	0.0417	#NUM!	CYCS
RNA Polym	6.760830E-02	1.17	0.0134	#NUM!	NELFB,SRSF11
Semaphori	6.918310E-02	1.16	0.0133	#NUM!	ITGB4,PRKAR2A
Insulin pro	6.918310E-02	1.16	0.04	#NUM!	KIF5B
D-myo-ino	7.244360E-02	1.14	0.0385	#NUM!	PIP4K2A
Syndecan i	7.413102E-02	1.13	0.037	#NUM!	ITGB4
Pyroptosis	7.413102E-02	1.13	0.037	#NUM!	CYCS
Mitochonc	7.585776E-02	1.12	0.00872	#NUM!	CYCS,GPD2,PRKAR2A
Glyoxylate	7.762471E-02	1.11	0.0357	#NUM!	GOT2
CDP-diacyl	7.762471E-02	1.11	0.0357	#NUM!	GPD2
Inhibition c	7.943282E-02	1.1	0.0123	#NUM!	PRKAR2A,PSMA2
Phagosom	7.943282E-02	1.1	0.0122	#NUM!	CTSB,HLA-B
Regulation	8.317638E-02	1.08	0.0333	#NUM!	PIP4K2A
Phosphatic	8.317638E-02	1.08	0.0333	#NUM!	GPD2
Role of p14	8.317638E-02	1.08	0.0333	#NUM!	NPM1
CDC42 Sigr	8.317638E-02	1.08	0.00694	#NUM!	ARPC4,CLIP1,HLA-B,ITGB4
RHO GTPa	8.511380E-02	1.07	0.0323	#NUM!	CLIP1
Sonic Hedg	8.511380E-02	1.07	0.0323	#NUM!	PRKAR2A
Protein Sor	9.120108E-02	1.04	0.0112	#NUM!	PRKAR2A,SERPINH1
Antimicrob	9.332543E-02	1.03	0.0294	#NUM!	S100A8
Late endos	9.332543E-02	1.03	0.0294	#NUM!	PLIN3
RHO GTPa	9.772372E-02	1.01	0.0278	#NUM!	ARPC4
Transcripti	9.772372E-02	1.01	0.0278	#NUM!	NPM1
Acute Phas	9.772372E-02	1.01	0.0108	#NUM!	C4A/C4B,SAA1
TWEAK Sig	1.004616E-01	0.998	0.027	#NUM!	CYCS
Compleme	1.004616E-01	0.998	0.027	#NUM!	C4A/C4B
Superpath	1.056818E-01	0.976	0.0256	#NUM!	GOT2
Antigen Pr	1.056818E-01	0.976	0.0256	#NUM!	HLA-B
Pyrimidine	1.081434E-01	0.966	0.025	#NUM!	NME1
DAG and IF	1.106624E-01	0.956	0.0244	#NUM!	PRKAR2A
TCF depen	1.106624E-01	0.956	0.0101	#NUM!	CUL3,PSMA2
MyD88:MA	1.132400E-01	0.946	0.0238	#NUM!	S100A8

DAP12 inte	1.132400E-01	0.946	0.0238	#NUM!	HLA-B
Formation	1.132400E-01	0.946	0.0238	#NUM!	HCFC1
Ephrin Rec	1.135011E-01	0.945	0.0099	#NUM!	ARPC4,ITGB4
Intra-Golgi	1.145513E-01	0.941	0.00985	#NUM!	KIF5B,PLIN3
Mitotic Prc	1.145513E-01	0.941	0.00985	#NUM!	CLIP1,NUMA1
Coronaviru	1.153453E-01	0.938	0.0098	#NUM!	NPM1,RPS3A
Vasopressi	1.158777E-01	0.936	0.0233	#NUM!	PRKAR2A
Smooth M	1.158777E-01	0.936	0.0233	#NUM!	CALD1
Pyrimidine	1.158777E-01	0.936	0.0233	#NUM!	NME1
Elastic fibr	1.183042E-01	0.927	0.0227	#NUM!	EFEMP1
SUMOylati	1.183042E-01	0.927	0.0227	#NUM!	NPM1
TAK1-depe	1.183042E-01	0.927	0.0227	#NUM!	SAA1
Regulation	1.183042E-01	0.927	0.0227	#NUM!	PABPC1
GPER1 sigr	1.207814E-01	0.918	0.0222	#NUM!	PRKAR2A
Apelin Pan	1.233105E-01	0.909	0.0217	#NUM!	PRKAR2A
ERK/MAPK	1.256030E-01	0.901	0.0093	#NUM!	ITGB4,PRKAR2A
TP53 Regu	1.309182E-01	0.883	0.0204	#NUM!	NPM1
Nucleosom	1.309182E-01	0.883	0.0204	#NUM!	NPM1
PFKFB4 Sig	1.309182E-01	0.883	0.0204	#NUM!	PRKAR2A
TNFR1 Sigr	1.358313E-01	0.867	0.0196	#NUM!	CYCS
Amyloid Pr	1.358313E-01	0.867	0.0196	#NUM!	PRKAR2A
Phagosom	1.377209E-01	0.861	0.00575	2	ARPC4,CLIP1,ITGB4,PIP4K2A
Apoptotic	1.383566E-01	0.859	0.0192	#NUM!	SPTAN1
Regulation	1.406048E-01	0.852	0.0189	#NUM!	PSMA2
Signaling b	1.406048E-01	0.852	0.0189	#NUM!	CGN
Phototrans	1.432188E-01	0.844	0.0185	#NUM!	PRKAR2A
Mitotic Me	1.448772E-01	0.839	0.00851	#NUM!	CLIP1,PSMA2
Intrinsic Pa	1.455459E-01	0.837	0.0182	#NUM!	CYCS
Deadenyla	1.455459E-01	0.837	0.0182	#NUM!	PABPC1
E3 ubiquiti	1.455459E-01	0.837	0.0182	#NUM!	HLA-B
Lymphoto›	1.455459E-01	0.837	0.0182	#NUM!	CYCS
Sertoli Cell	1.458814E-01	0.836	0.00847	#NUM!	ARPC4,PRKAR2A
Osteoarthr	1.458814E-01	0.836	0.00847	#NUM!	ITGB4,S100A8
NRF2-med	1.468926E-01	0.833	0.00844	#NUM!	CUL3,PPIB
Orexin Sigr	1.479108E-01	0.83	0.0084	#NUM!	PIP4K2A,PRKAR2A
CD27 Signa	1.506607E-01	0.822	0.0175	#NUM!	CYCS
FAT10 Sign	1.506607E-01	0.822	0.0175	#NUM!	PSMA2
Sertoli Cell	1.531087E-01	0.815	0.00823	#NUM!	CGN,SPTAN1
Actin Cytos	1.538155E-01	0.813	0.0082	#NUM!	ARPC4,ITGB4
Metabolisr	1.552387E-01	0.809	0.0169	#NUM!	PDXK
Metabolisr	1.552387E-01	0.809	0.0169	#NUM!	PSMA2
Neddylatio	1.559553E-01	0.807	0.00813	#NUM!	CUL3,PSMA2
Pancreatic	1.570363E-01	0.804	0.0081	#NUM!	CA1,PRKAR2A
NIK-->nonc	1.577611E-01	0.802	0.0167	#NUM!	PSMA2
Retinoic ac	1.577611E-01	0.802	0.0167	#NUM!	CYCS
SPINK1 Par	1.577611E-01	0.802	0.0167	#NUM!	CTSB
Docosahex	1.599558E-01	0.796	0.008	#NUM!	CYCS,PRKAR2A
Collagen d	1.603245E-01	0.795	0.0164	#NUM!	CTSB

NCAM sign	1.603245E-01	0.795	0.0164	#NUM!	SPTAN1
Kinesins	1.603245E-01	0.795	0.0164	#NUM!	KIF5B
Cytoprotec	1.625549E-01	0.789	0.0161	#NUM!	CYCS
Hedgehog	1.698244E-01	0.77	0.0154	#NUM!	PSMA2
PXR/RXR A	1.698244E-01	0.77	0.0154	#NUM!	PRKAR2A
Activation	1.698244E-01	0.77	0.0154	#NUM!	PPIB
Induction c	1.698244E-01	0.77	0.0154	#NUM!	CYCS
Pyridoxal 5	1.698244E-01	0.77	0.0154	#NUM!	PDXK
Deubiquiti	1.729816E-01	0.762	0.0076	#NUM!	HCFC1,PSMA2
TNFR2 non	1.770109E-01	0.752	0.0147	#NUM!	PSMA2
Axonal Gui	1.786488E-01	0.748	0.00588	#NUM!	ARPC4,ITGB4,PRKAR2A
Agrin Inter	1.790606E-01	0.747	0.0145	#NUM!	CTTN
TP53 Regu	1.815516E-01	0.741	0.0143	#NUM!	NELFB
Cell Cycle (	1.823896E-01	0.739	0.00735	#NUM!	CLIP1,PSMA2
Interferon	1.840772E-01	0.735	0.0141	#NUM!	HLA-B
COPII-med	1.862087E-01	0.73	0.0139	#NUM!	USO1
Melatonin	1.862087E-01	0.73	0.0139	#NUM!	PRKAR2A
Signaling b	1.887991E-01	0.724	0.0137	#NUM!	HNRNPM
Regulation	1.887991E-01	0.724	0.0137	#NUM!	PSMA2
Amyloid fil	1.887991E-01	0.724	0.0137	#NUM!	SAA1
Granzyme	1.909853E-01	0.719	0.0135	#NUM!	NME1
Cellular res	1.931968E-01	0.714	0.0133	#NUM!	PSMA2
GPCR-Med	1.931968E-01	0.714	0.0133	#NUM!	PRKAR2A
Huntingtor	1.945360E-01	0.711	0.00704	#NUM!	CYCS,PSMA2
Leptin Sign	1.954339E-01	0.709	0.0132	#NUM!	PRKAR2A
Macropino	1.954339E-01	0.709	0.0132	#NUM!	ITGB4
DDX58/IFII	2.023019E-01	0.694	0.0127	#NUM!	SAA1
Glucose me	2.046445E-01	0.689	0.0125	#NUM!	GOT2
Dopamine	2.046445E-01	0.689	0.0125	#NUM!	PRKAR2A
PI Metabol	2.094112E-01	0.679	0.0122	#NUM!	PIP4K2A
Signaling b	2.113489E-01	0.675	0.012	#NUM!	PSMA2
Transcripti	2.137962E-01	0.67	0.0119	#NUM!	ADIRF
Activation	2.137962E-01	0.67	0.0119	#NUM!	PRKAR2A
Autism Sig	2.197860E-01	0.658	0.00649	#NUM!	HLA-B,PRKAR2A
IL-17A Sigr	2.208005E-01	0.656	0.0115	#NUM!	SERPINH1
Regulation	2.228435E-01	0.652	0.0114	#NUM!	PSMA2
TP53 Regu	2.228435E-01	0.652	0.0114	#NUM!	CYCS
Synaptoge	2.269865E-01	0.644	0.00635	#NUM!	ARPC4,PRKAR2A
Opioid Sigr	2.269865E-01	0.644	0.0111	#NUM!	PRKAR2A
Regulation	2.269865E-01	0.644	0.0111	#NUM!	ITGB4
Unfolded p	2.269865E-01	0.644	0.0111	#NUM!	PDIA6
Ceramide S	2.296149E-01	0.639	0.011	#NUM!	CYCS
Crosstalk b	2.296149E-01	0.639	0.011	#NUM!	HLA-B
BMP signal	2.296149E-01	0.639	0.011	#NUM!	PRKAR2A
Apelin Adip	2.296149E-01	0.639	0.011	#NUM!	PRKAR2A
Degradatic	2.317395E-01	0.635	0.0109	#NUM!	PSMA2
Cell junctic	2.317395E-01	0.635	0.0109	#NUM!	ITGB4
MAPK6/MA	2.317395E-01	0.635	0.0109	#NUM!	PSMA2

Pyroptosis	2.360478E-01	0.627	0.0106	#NUM!	PRKAR2A
EPH-Ephrir	2.360478E-01	0.627	0.0106	#NUM!	ARPC4
Interferon	2.360478E-01	0.627	0.0106	#NUM!	HLA-B
Fcγ Recept	2.360478E-01	0.627	0.0106	#NUM!	ARPC4
Glutamine	2.376840E-01	0.624	0.00615	#NUM!	GOT2,PRKAR2A
Gap Juncti	2.376840E-01	0.624	0.00615	#NUM!	CTTN,PRKAR2A
Myelinatio	2.398833E-01	0.62	0.00612	#NUM!	ITGB4,PRKAR2A
Transcripti	2.404363E-01	0.619	0.0104	#NUM!	PSMA2
Small Cell I	2.404363E-01	0.619	0.0104	#NUM!	CYCS
IL-1 Signali	2.404363E-01	0.619	0.0104	#NUM!	PRKAR2A
Cellular res	2.426610E-01	0.615	0.0103	#NUM!	SERPINH1
Salvage Pa	2.426610E-01	0.615	0.0103	#NUM!	NME1
Protein fol	2.449063E-01	0.611	0.0102	#NUM!	TBCA
Melanocyt	2.449063E-01	0.611	0.0102	#NUM!	PRKAR2A
UVA-Induc	2.449063E-01	0.611	0.0102	#NUM!	CYCS
S Phase	2.488857E-01	0.604	0.01	#NUM!	PSMA2
ABC-family	2.511886E-01	0.6	0.0099	#NUM!	PSMA2
Neuropath	2.511886E-01	0.6	0.0099	#NUM!	PRKAR2A
DNA Replic	2.576321E-01	0.589	0.00962	#NUM!	PSMA2
IGF-1 Signa	2.600160E-01	0.585	0.00952	#NUM!	PRKAR2A
WNK Rena	2.618183E-01	0.582	0.00943	#NUM!	CUL3
Paxillin Sig	2.642409E-01	0.578	0.00935	#NUM!	ITGB4
PD-1, PD-L	2.642409E-01	0.578	0.00935	#NUM!	HLA-B
α-Adrenerg	2.685344E-01	0.571	0.00917	#NUM!	PRKAR2A
Interleukin	2.703958E-01	0.568	0.00909	#NUM!	SAA1
Sleep REM	2.722701E-01	0.565	0.00901	#NUM!	PRKAR2A
Binding an	2.747894E-01	0.561	0.00893	#NUM!	SAA1
Oxidative F	2.747894E-01	0.561	0.00893	#NUM!	CYCS
Sleep NREI	2.805434E-01	0.552	0.0087	#NUM!	PRKAR2A
CDK5 Signa	2.805434E-01	0.552	0.0087	#NUM!	PRKAR2A
Amyotroph	2.831392E-01	0.548	0.00862	#NUM!	CYCS
Cachexia S	2.831392E-01	0.548	0.00543	#NUM!	PRKAR2A,PSMA2
Neuregulir	2.851018E-01	0.545	0.00855	#NUM!	ITGB4
PAK Signali	2.851018E-01	0.545	0.00855	#NUM!	ITGB4
GPCR-Med	2.870781E-01	0.542	0.00847	#NUM!	PRKAR2A
Synthesis c	2.890680E-01	0.539	0.0084	#NUM!	PSMA2
Nitric Oxid	2.910717E-01	0.536	0.00833	#NUM!	PRKAR2A
Renin-Angi	2.930893E-01	0.533	0.00826	#NUM!	PRKAR2A
Th1 Pathw	2.951209E-01	0.53	0.0082	#NUM!	HLA-B
L1CAM int	3.013006E-01	0.521	0.008	#NUM!	SPTAN1
Gαs Signali	3.033891E-01	0.518	0.00794	#NUM!	PRKAR2A
Endocanna	3.054921E-01	0.515	0.00787	#NUM!	PRKAR2A
Electron tr	3.069022E-01	0.513	0.00781	#NUM!	CYCS
IL-27 Signa	3.069022E-01	0.513	0.00781	#NUM!	HLA-B
Interleukin	3.090295E-01	0.51	0.00775	#NUM!	PSMA2
G Beta Gar	3.090295E-01	0.51	0.00775	#NUM!	PRKAR2A
Mitotic G1	3.133286E-01	0.504	0.00763	#NUM!	PSMA2
fMLP Signa	3.133286E-01	0.504	0.00763	#NUM!	ARPC4

Ferroptosis	3.133286E-01	0.504	0.00763	#NUM!	CTSB
Synaptic Lo	3.147748E-01	0.502	0.00758	#NUM!	PRKAR2A
HGF Signal	3.147748E-01	0.502	0.00758	#NUM!	ITGB4
Atheroscle	3.169567E-01	0.499	0.00752	#NUM!	S100A8
P2Y Purige	3.169567E-01	0.499	0.00752	#NUM!	PRKAR2A
Compleme	3.206269E-01	0.494	0.00741	#NUM!	C4A/C4B
SNARE Sigr	3.228494E-01	0.491	0.00735	#NUM!	PRKAR2A
Th2 Pathw	3.250873E-01	0.488	0.0073	#NUM!	HLA-B
Reelin Sign	3.265878E-01	0.486	0.00725	#NUM!	ARPC4
White Adip	3.265878E-01	0.486	0.00725	#NUM!	PRKAR2A
RHO GTPas	3.288516E-01	0.483	0.00719	#NUM!	CLIP1
PIP3 activa	3.303695E-01	0.481	0.00714	#NUM!	PIP4K2A
Gαi Signali	3.303695E-01	0.481	0.00714	#NUM!	PRKAR2A
Insulin Rec	3.303695E-01	0.481	0.00714	#NUM!	PRKAR2A
MSP-RON S	3.303695E-01	0.481	0.00714	#NUM!	ITGB4
Hereditary	3.341950E-01	0.476	0.00704	#NUM!	NPM1
Xenobiotic	3.365116E-01	0.473	0.00699	#NUM!	CUL3
C-type lect	3.404082E-01	0.468	0.0069	#NUM!	PSMA2
Hepatic Fib	3.411929E-01	0.467	0.00473	#NUM!	ITGB4,PRKAR2A
Cytotoxic T	3.427678E-01	0.465	0.00471	#NUM!	CYCS,HLA-B
Endocanna	3.443499E-01	0.463	0.0068	#NUM!	PRKAR2A
WNT/SHH	3.475362E-01	0.459	0.00671	#NUM!	PRKAR2A
Endocanna	3.475362E-01	0.459	0.00671	#NUM!	PRKAR2A
Dilated Car	3.499452E-01	0.456	0.00667	#NUM!	PRKAR2A
PTEN Regu	3.499452E-01	0.456	0.00667	#NUM!	PSMA2
Cellular Eff	3.499452E-01	0.456	0.00667	#NUM!	PRKAR2A
PTEN Signa	3.515604E-01	0.454	0.00662	#NUM!	ITGB4
Transcripti	3.531832E-01	0.452	0.00658	#NUM!	PSMA2
Corticotrop	3.531832E-01	0.452	0.00658	#NUM!	PRKAR2A
IL-10 Signa	3.572728E-01	0.447	0.00649	#NUM!	HLA-B
Relaxin Sig	3.589219E-01	0.445	0.00645	#NUM!	PRKAR2A
eNOS Signa	3.605786E-01	0.443	0.00641	#NUM!	PRKAR2A
Fcgamma r	3.622430E-01	0.441	0.00637	#NUM!	ARPC4
Ovarian Ca	3.647539E-01	0.438	0.00633	#NUM!	PRKAR2A
Epithelial A	3.647539E-01	0.438	0.00633	#NUM!	ARPC4
Microauto	3.681290E-01	0.434	0.00625	#NUM!	PSMA2
HEY1 Signa	3.698282E-01	0.432	0.00621	#NUM!	NELFB
Androgen S	3.845918E-01	0.415	0.00592	#NUM!	PRKAR2A
Signaling b	3.863670E-01	0.413	0.00588	#NUM!	PSMA2
Aldosteror	3.899420E-01	0.409	0.00581	#NUM!	PIP4K2A
Th1 and Th	3.899420E-01	0.409	0.00581	#NUM!	HLA-B
G alpha (q)	3.926449E-01	0.406	0.00575	#NUM!	SAA1
Tumor Mic	4.017908E-01	0.396	0.00559	#NUM!	HLA-B
Cardiac β-a	4.036454E-01	0.394	0.00556	#NUM!	PRKAR2A
BBSome Si	4.083194E-01	0.389	0.00409	#NUM!	CUL3,PRKAR2A
IL-33 Signa	4.120975E-01	0.385	0.00541	#NUM!	PRKAR2A
Dopamine-	4.139997E-01	0.383	0.00538	#NUM!	PRKAR2A
GNRH Sign	4.216965E-01	0.375	0.00524	#NUM!	PRKAR2A

Productior	4.216965E-01	0.375	0.00524	#NUM!	S100A8
Leukocyte	4.255984E-01	0.371	0.00518	#NUM!	CTTN
Acetylcholi	4.265795E-01	0.37	0.00515	#NUM!	PRKAR2A
Type I Diak	4.285485E-01	0.368	0.00393	#NUM!	CYCS,HLA-B
Xenobiotic	4.285485E-01	0.368	0.00513	#NUM!	PRKAR2A
NUR77 Sigı	4.325138E-01	0.364	0.0039	#NUM!	CYCS,HLA-B
Natural Kil	4.335109E-01	0.363	0.00505	#NUM!	HLA-B
D-myo-ino	4.335109E-01	0.363	0.00505	#NUM!	PIP4K2A
Mitotic G2	4.355119E-01	0.361	0.00503	#NUM!	PSMA2
Adrenome	4.355119E-01	0.361	0.00503	#NUM!	PRKAR2A
CD28 Signa	4.365158E-01	0.36	0.00387	#NUM!	ARPC4,HLA-B
Adrenergic	4.365158E-01	0.36	0.005	#NUM!	PRKAR2A
PI3K/AKT S	4.365158E-01	0.36	0.005	#NUM!	ITGB4
ID1 Signalii	4.385307E-01	0.358	0.00498	#NUM!	CUL3
ILK Signalir	4.385307E-01	0.358	0.00498	#NUM!	ITGB4
CDX Gastro	4.405549E-01	0.356	0.00495	#NUM!	CA1
Immunore	4.405549E-01	0.356	0.00495	#NUM!	HLA-B
Gustation I	4.405549E-01	0.356	0.00495	#NUM!	PRKAR2A
Molecular	4.436086E-01	0.353	0.0035	#NUM!	CYCS,ITGB4,PRKAR2A
Fc epsilon	4.466836E-01	0.35	0.00485	#NUM!	PSMA2
Role of Tis	4.477133E-01	0.349	0.00483	#NUM!	PDIA6
3-phospho	4.497799E-01	0.347	0.00481	#NUM!	PIP4K2A
IL-8 Signali	4.528976E-01	0.344	0.00476	#NUM!	LASP1
Keratinizat	4.591980E-01	0.338	0.00467	#NUM!	EVPL
mTOR Sign	4.591980E-01	0.338	0.00467	#NUM!	RPS3A
Cardiac Hy	4.602566E-01	0.337	0.00369	#NUM!	ITGB4,PRKAR2A
Autophagy	4.634469E-01	0.334	0.00461	#NUM!	PRKAR2A
Calcium Sig	4.688134E-01	0.329	0.00455	#NUM!	PRKAR2A
Multiple Sc	4.709773E-01	0.327	0.0045	#NUM!	HLA-B
Hepatic Ch	4.731513E-01	0.325	0.00448	#NUM!	PRKAR2A
Role of NF	4.742420E-01	0.324	0.00446	#NUM!	PRKAR2A
NAFLD Sigr	4.775293E-01	0.321	0.00442	#NUM!	CTSB
HER-2 Sign	4.786301E-01	0.32	0.00441	#NUM!	ITGB4
Neurovasc	4.864072E-01	0.313	0.00431	#NUM!	PRKAR2A
IL-12 Signa	4.909079E-01	0.309	0.00426	#NUM!	S100A8
Superpath	4.920395E-01	0.308	0.00424	#NUM!	PIP4K2A
cAMP-mec	4.920395E-01	0.308	0.00424	#NUM!	PRKAR2A
AMPK Sign	5.011872E-01	0.3	0.00413	#NUM!	PRKAR2A
Role of Ost	5.035006E-01	0.298	0.0041	#NUM!	CTSB
Wound He	5.152286E-01	0.288	0.00397	#NUM!	ITGB4
Sperm Mo	5.223962E-01	0.282	0.00389	#NUM!	PRKAR2A
G alpha (i)	5.236004E-01	0.281	0.00388	#NUM!	SAA1
Cardiac Hy	5.272299E-01	0.278	0.00383	#NUM!	PRKAR2A
Circadian F	5.370318E-01	0.27	0.00373	#NUM!	PRKAR2A
Colorectal	5.407543E-01	0.267	0.00369	#NUM!	PRKAR2A
Chaperone	5.445027E-01	0.264	0.00315	#NUM!	PLIN3,PSMA2
Opioid Sigr	5.533501E-01	0.257	0.00357	#NUM!	PRKAR2A
Eicosanoid	5.533501E-01	0.257	0.00357	#NUM!	PRKAR2A

Oxytocin S	5.559043E-01	0.255	0.00355	#NUM!	PRKAR2A
CLEAR Sigr	5.597576E-01	0.252	0.00351	#NUM!	CTSB
Sirtuin Sigr	5.675446E-01	0.246	0.00344	#NUM!	GOT2
Xenobiotic	5.701643E-01	0.244	0.0034	#NUM!	CUL3
Senescenc	5.767665E-01	0.239	0.00334	#NUM!	SAA1
Class A/1 (	5.984116E-01	0.223	0.00315	#NUM!	SAA1
Neuroinfla	5.984116E-01	0.223	0.00315	#NUM!	HLA-B
Glucocorti	1.000000E+00	0	0.00172	#NUM!	HLA-B
S100 Famil	1.000000E+00	0	0.00259	#NUM!	PRKAR2A,S100A8
RAR Activa	1.000000E+00	0	0.00233	#NUM!	PRKAR2A
Role of NF	1.000000E+00	0	0.000967	#NUM!	HLA-B
Calcium-in	1.000000E+00	0	0.00217	#NUM!	HLA-B
CTLA4 Sign	1.000000E+00	0	0.00164	#NUM!	HLA-B
T Helper C	1.000000E+00	0	0.00212	#NUM!	HLA-B
Dendritic C	1.000000E+00	0	0.00168	#NUM!	HLA-B
ICOS-ICOSI	1.000000E+00	0	0.00197	#NUM!	HLA-B
CREB Signa	1.000000E+00	0	0.00165	#NUM!	PRKAR2A
Allograft R	1.000000E+00	0	0.00205	#NUM!	HLA-B
Autoimmu	1.000000E+00	0	0.00218	#NUM!	HLA-B
Graft-versu	1.000000E+00	0	0.00224	#NUM!	HLA-B
Communic	1.000000E+00	0	0.00107	#NUM!	HLA-B
Systemic L	1.000000E+00	0	0.000938	#NUM!	HLA-B
FAK Signali	1.000000E+00	0	0.00192	#NUM!	ARPC4,ITGB4
Phospholip	1.000000E+00	0	0.000893	#NUM!	ITGB4
Altered T C	1.000000E+00	0	0.00107	#NUM!	HLA-B
Protein Kir	1.000000E+00	0	0.00243	#NUM!	PRKAR2A
B Cell Deve	1.000000E+00	0	0.00205	#NUM!	HLA-B
Breast Can	1.000000E+00	0	0.00168	#NUM!	PRKAR2A
PKCθ Signa	1.000000E+00	0	0.00179	#NUM!	HLA-B
Antiprolife	1.000000E+00	0	0.00235	#NUM!	PABPC1
OX40 Signa	1.000000E+00	0	0.00209	#NUM!	HLA-B
TEC Kinase	1.000000E+00	0	0.00173	#NUM!	ITGB4
Estrogen R	1.000000E+00	0	0.00244	#NUM!	PRKAR2A
Serotonin	1.000000E+00	0	0.00213	#NUM!	PRKAR2A
T Cell Rece	1.000000E+00	0	0.00162	#NUM!	HLA-B
G-Protein (	1.000000E+00	0	0.00142	#NUM!	PRKAR2A
T Cell Exha	1.000000E+00	0	0.00176	#NUM!	HLA-B
Systemic L	1.000000E+00	0	0.00156	#NUM!	HLA-B

MA2,S100A8,SPTAN1,SRP14

Supplement table 2. Diferentially expressed proteins in IPF lung tissues

	FC	log2(FC)	raw.pval	#NAME?
PLIN3	113.8	6.8303	1.34E-05	4.8713
HCFC1	0.021301	-5.553	3.86E-05	4.4137
SRSF11	10.934	3.4507	0.000558	3.253
GOT2	69.792	6.125	0.000924	3.0344
PIP4K2A	28.799	4.848	0.001396	2.855
PDIA6	11.933	3.5769	0.001752	2.7564
SERPINH1	8.6939	3.12	0.001753	2.7562
ERC1	60.984	5.9304	0.002596	2.5857
TBCA	8.4263	3.0749	0.00287	2.5421
ARPC4	18.199	4.1858	0.003097	2.509
CSRP2	0.044171	-4.5008	0.003371	2.4723
PABPC1	17.825	4.1558	0.003699	2.432
ACADVL	0.076888	-3.7011	0.003862	2.4132
PRDX3	3.4105	1.77	0.003918	2.4069
NUMA1	0.17715	-2.497	0.004251	2.3715
CTTN	0.073382	-3.7684	0.004434	2.3532
BZW1	7.0332	2.8142	0.00477	2.3215
PDLIM2	0.03152	-4.9876	0.005138	2.2892
NID2	0.018354	-5.7678	0.005351	2.2716
PGM1	27.64	4.7887	0.006797	2.1677
CAVIN2	0.30732	-1.7022	0.008871	2.052
RPL38	5.7121	2.514	0.00984	2.007
NELFB	62.909	5.9752	0.010233	1.99
PPIB	7.6251	2.9308	0.010747	1.9687
S100A8	5.9271	2.5673	0.011707	1.9315
CEP170	11.46	3.5185	0.011912	1.924
ADIRF	0.089893	-3.4756	0.012662	1.8975
HECTD1	6.5128	2.7033	0.014883	1.8273
DCPS	13.537	3.7588	0.015703	1.804
HINT2	0.16119	-2.6331	0.016519	1.782
SRP14	0.18806	-2.4107	0.021065	1.6764
CLIC5	0.044844	-4.4789	0.021484	1.6679
SPTAN1	0.42669	-1.2287	0.022095	1.6557
EVPL	0.23867	-2.0669	0.022314	1.6514
HNMT	0.18029	-2.4716	0.022659	1.6448
PDXK	8.1612	3.0288	0.023942	1.6208
SAA1	10.613	3.4078	0.025799	1.5884
SMTN	0.10217	-3.291	0.026498	1.5768
PLIN4	0.074885	-3.7392	0.02654	1.5761
CGN	0.052645	-4.2476	0.02661	1.5749
HLA-B	7.8322	2.9694	0.02871	1.542
ITGB4	10.055	3.3298	0.030233	1.5195
HNRNPA3	0.39674	-1.3337	0.031724	1.4986
LASP1	0.28473	-1.8123	0.031765	1.4981
KIF5B	0.29463	-1.763	0.032184	1.4924

CTSB	5.132	2.3595	0.032557	1.4874
HNRNPM	4.6959	2.2314	0.033903	1.4698
RPS3A	0.31807	-1.6526	0.035262	1.4527
PURB	0.15649	-2.6758	0.035939	1.4444
EHD2	0.21471	-2.2196	0.037727	1.4234
NME1	4.1509	2.0534	0.037753	1.423
CLIP1	3.0146	1.592	0.037842	1.422
MVP	3.6004	1.8482	0.03794	1.4209
PSMA2	52.036	5.7014	0.039768	1.4005
CYCS	11.512	3.5251	0.040542	1.3921
C4A	2.2781	1.1878	0.040588	1.3916
C3(1)	0.31969	-1.6453	0.041409	1.3829
NPM1	4.2676	2.0934	0.041662	1.3803
GPD2	0.34412	-1.539	0.041776	1.3791
TALDO1	2.4325	1.2824	0.042361	1.373
DDRGK1	3.4888	1.8027	0.042875	1.3678
EFEMP1	0.23736	-2.0749	0.044248	1.3541
ENAH	0.22794	-2.1333	0.044267	1.3539
CUL3	0.2669	-1.9057	0.044506	1.3516
SEPTIN9	0.22738	-2.1368	0.045621	1.3408
CA1	2.1825	1.126	0.046374	1.3337
CALD1	0.33731	-1.5679	0.046733	1.3304
PRKAR2A	0.69019	-0.53493	0.047919	1.3195
USO1	0.36936	-1.4369	0.049117	1.3088
RPL12	3.0019	1.5859	0.049173	1.3083

Supplement table 3. IPA pathway analysis summary of differentially expressed proteins in IPF BALF
© 2000-2024 QIAGEN. All rights reserved.

Ingenuity (	p-val	-log(p-valu	Ratio	z-score	Molecules
Compleme	3.01995E-07	6.52	0.0519	#NUM!	C8G,CPN1,IGHV3-13,IGHV3-7,IGHV7-81,IGK\
Neutrophil	3.38844E-06	5.47	0.021	0	CAB39,CTSC,GM2A,HK3,IST1,JUP,PRG2,SERP
B Cell Deve	4.2658E-06	5.37	0.0205	#NUM!	IGHV1-3,IGHV3-13,IGHV3-20,IGHV3-64,IGHV
B Cell Rece	6.60693E-06	5.18	0.0174	#NUM!	CDC42,IGHV1-3,IGHV3-13,IGHV3-20,IGHV3-(
IL-15 Signa	8.12831E-06	5.09	0.019	#NUM!	IGHV1-3,IGHV3-13,IGHV3-20,IGHV3-64,IGHV
FcγRIIB Sig	8.91251E-06	5.05	0.0188	#NUM!	IGHV1-3,IGHV3-13,IGHV3-20,IGHV3-64,IGHV
Fcgamma r	1.28825E-05	4.89	0.0382	-0.816	CDC42,IGHV3-13,IGHV3-7,IGHV7-81,IGKV1D
p70S6K Sig	1.86209E-05	4.73	0.0173	#NUM!	IGHV1-3,IGHV3-13,IGHV3-20,IGHV3-64,IGHV
PI3K Signal	2.18776E-05	4.66	0.017	#NUM!	IGHV1-3,IGHV3-13,IGHV3-20,IGHV3-64,IGHV
Binding an	3.38844E-05	4.47	0.0446	-0.447	IGHV3-13,IGHV3-7,IGHV7-81,IGKV1D-16,IGL
Fc epsilon	5.88844E-05	4.23	0.0291	-0.816	IGHV3-13,IGHV3-7,IGHV7-81,IGKV1D-16,IGL
Systemic L	0.000120226	3.92	0.0138	#NUM!	IGHV1-3,IGHV3-13,IGHV3-20,IGHV3-64,IGHV
Signaling b	0.000239883	3.62	0.0294	-0.447	IGHV3-13,IGHV3-7,IGHV7-81,IGKV1D-16,IGL
Prostanoid	0.000467735	3.33	0.2	#NUM!	PTGDS,PTGES3
Immunore	0.000524807	3.28	0.0248	-0.447	IGHV3-13,IGHV3-7,IGHV7-81,IGKV1D-16,IGL
GDP-gluco	0.00057544	3.24	0.182	#NUM!	HK3,PGM3
Airway Pat	0.000616595	3.21	0.0339	#NUM!	APOM,C8G,LCN1,PTGDS
Cell surfac	0.000645654	3.19	0.0237	-0.447	IGHV3-13,IGHV3-7,IGHV7-81,IGKV1D-16,IGL
Systemic L	0.000676083	3.17	0.0103	#NUM!	C8G,IGHV1-3,IGHV3-13,IGHV3-20,IGHV3-64,
Glucose an	0.000676083	3.17	0.167	#NUM!	HK3,PGM3
Guanosine	0.000812831	3.09	0.154	#NUM!	NT5C3B,NT5E
UDP-N-ace	0.000812831	3.09	0.154	#NUM!	HK3,PGM3
Communic	0.000891251	3.05	0.0107	#NUM!	IGHV1-3,IGHV3-13,IGHV3-20,IGHV3-64,IGHV
Urate Bios	0.000933254	3.03	0.143	#NUM!	NT5C3B,NT5E
Colanic Aci	0.000933254	3.03	0.143	#NUM!	UGDH,UGP2
Altered T C	0.000933254	3.03	0.0107	#NUM!	IGHV1-3,IGHV3-13,IGHV3-20,IGHV3-64,IGHV
Phospholip	0.001023293	2.99	0.00982	#NUM!	CDC42,IGHV1-3,IGHV3-13,IGHV3-20,IGHV3-(
Synthesis c	0.001071519	2.97	0.133	#NUM!	PTGDS,PTGES3
Adenosine	0.001230269	2.91	0.125	#NUM!	NT5C3B,NT5E
Remodelin	0.00144544	2.84	0.0441	#NUM!	ACTN1,NME1,TUBB
Purine Nuc	0.001737801	2.76	0.105	#NUM!	NT5C3B,NT5E
Role of NF/	0.001949845	2.71	0.00967	#NUM!	IGHV1-3,IGHV3-13,IGHV3-20,IGHV3-64,IGHV
Macropinc	0.001995262	2.7	0.0395	#NUM!	ANKFY1,CDC42,CSF1R
Interconve	0.002344229	2.63	0.0909	#NUM!	GSR,NME1
Germ Cell-	0.002344229	2.63	0.0235	#NUM!	ACTN1,CDC42,JUP,TUBB
Pyrimidine	0.002570396	2.59	0.087	#NUM!	NME1,NME7
NAD Salvag	0.003548134	2.45	0.0741	#NUM!	NT5C3B,NT5E
RHO GTPa:	0.003548134	2.45	0.0133	0.816	ACTN1,ANKFY1,CDC42,JUP,OBSCN,USP9X
UDP-D-xyl(	0.006456542	2.19	0.5	#NUM!	UGDH
Pyrimidine	0.007585776	2.12	0.05	#NUM!	NME1,NME7
Sertoli Cell	0.008317638	2.08	0.0165	0	ACTN1,CDC42,JUP,TUBB
Pyrimidine	0.008709636	2.06	0.0465	#NUM!	NME1,NME7
Glutathion	0.012882496	1.89	0.25	#NUM!	GSR
Deadenyla	0.014125375	1.85	0.0364	#NUM!	EIF4A2,NT5C3B

Pentose Pł	0.016218101	1.79	0.2	#NUM!	PGLS
UDP-N-ace	0.019498446	1.71	0.167	#NUM!	PGM3
Phase II - C	0.020417379	1.69	0.0299	#NUM!	UGDH,UGP2
Aryl hydro	0.022908677	1.64	0.143	#NUM!	PTGES3
Trehalose	0.022908677	1.64	0.143	#NUM!	HK3
Glycogen E	0.022908677	1.64	0.143	#NUM!	UGP2
ISG15 anti	0.022908677	1.64	0.0282	#NUM!	EIF4A2,UBE2N
Mitotic Prc	0.028840315	1.54	0.0148	#NUM!	EML4,NME7,TUBB
Lipophagy	0.028840315	1.54	0.111	#NUM!	PLIN3
Clathrin-m	0.030902954	1.51	0.0144	#NUM!	APOM,CDC42,USP9X
Ketone boc	0.032359366	1.49	0.1	#NUM!	BDH2
RHOBTB3 /	0.032359366	1.49	0.1	#NUM!	PLIN3
Ketolysis	0.032359366	1.49	0.1	#NUM!	BDH2
RHO GTPa:	0.035481339	1.45	0.0909	#NUM!	CDC42
Ketogenes	0.035481339	1.45	0.0909	#NUM!	BDH2
Pentose Pł	0.035481339	1.45	0.0909	#NUM!	PGLS
Glycogen D	0.035481339	1.45	0.0909	#NUM!	PGM3
Acute Mye	0.035481339	1.45	0.022	#NUM!	CSF1R,JUP
NER (Nucle	0.035481339	1.45	0.022	#NUM!	COPS3,UBE2N
Cell junctic	0.036307805	1.44	0.0217	#NUM!	ACTN1,JUP
Nucleotide	0.039810717	1.4	0.0206	#NUM!	COPS3,UBE2N
Salvage Pa	0.039810717	1.4	0.0206	#NUM!	NME1,NME7
Processing	0.041686938	1.38	0.0769	#NUM!	PHB2
Transcripti	0.041686938	1.38	0.0769	#NUM!	PTGDS
Glycogen D	0.041686938	1.38	0.0769	#NUM!	PGM3
Cargo reco	0.047863009	1.32	0.0187	#NUM!	COPS3,SCARB2
Paxillin Sig	0.047863009	1.32	0.0187	#NUM!	ACTN1,CDC42
Signaling b	0.048977882	1.31	0.0185	#NUM!	CDC42,JUP
Chondroiti	0.051286138	1.29	0.0625	#NUM!	GM2A
Dermatan	0.05370318	1.27	0.0588	#NUM!	GM2A
ESR-media	0.057543994	1.24	0.0169	#NUM!	CARM1,PTGES3
RORA activ	0.057543994	1.24	0.0556	#NUM!	CARM1
Nicotinate	0.057543994	1.24	0.0556	#NUM!	NT5E
NFE2L2 reg	0.060255959	1.22	0.0526	#NUM!	GSR
IL-15 Produ	0.0616595	1.21	0.0163	#NUM!	CSF1R,PTK7
RHO GTPa:	0.066069345	1.18	0.0476	#NUM!	CDC42
Response t	0.069183097	1.16	0.0152	#NUM!	ACTN1,PLEK
Chaperone	0.069183097	1.16	0.0455	#NUM!	PLIN3
Nephrin fa	0.072443596	1.14	0.0435	#NUM!	ACTN1
Other inte	0.075857758	1.12	0.0417	#NUM!	CSF1R
Glycogen r	0.075857758	1.12	0.0417	#NUM!	UGP2
MyD88 cas	0.079432823	1.1	0.04	#NUM!	UBE2N
BMAL1:CL(	0.085113804	1.07	0.037	#NUM!	CARM1
Syndecan i	0.085113804	1.07	0.037	#NUM!	ACTN1
Glutathion	0.087096359	1.06	0.0357	#NUM!	GSR
Energy dep	0.091201084	1.04	0.0345	#NUM!	CAB39
Myogenesi	0.091201084	1.04	0.0345	#NUM!	CDC42

Protein ub	0.09332543	1.03	0.0333	#NUM!	USP9X
Nucleotide	0.09332543	1.03	0.0333	#NUM!	NT5E
Asparagine	0.095499259	1.02	0.0323	#NUM!	PGM3
RHO GTPas	0.095499259	1.02	0.0323	#NUM!	CDC42
Signaling b	0.095499259	1.02	0.0323	#NUM!	CSF1R
MyD88 dep	0.095499259	1.02	0.0323	#NUM!	UBE2N
Toll Like Re	0.1	1	0.0312	#NUM!	UBE2N
G-protein b	0.1	1	0.0312	#NUM!	CDC42
Phagosom	0.100693167	0.997	0.0122	#NUM!	CTSC,TUBB
Airway Infl	0.102565193	0.989	0.0303	#NUM!	PRG2
Cargo conc	0.10543869	0.977	0.0294	#NUM!	CTSC
Triglycerid	0.10543869	0.977	0.0294	#NUM!	PLIN3
Late endos	0.10543869	0.977	0.0294	#NUM!	PLIN3
GPVI-medi	0.108392691	0.965	0.0286	#NUM!	CDC42
RHO GTPas	0.111173173	0.954	0.0278	#NUM!	CDC42
MyD88-inc	0.114287833	0.942	0.027	#NUM!	UBE2N
Detoxificat	0.114287833	0.942	0.027	#NUM!	GSR
Gene and p	0.114287833	0.942	0.027	#NUM!	CDC42
Compleme	0.114287833	0.942	0.027	#NUM!	C8G
Protein Sor	0.115345326	0.938	0.0112	#NUM!	CDC42,SCARB2
MAP kinas	0.11994993	0.921	0.0256	#NUM!	UBE2N
Transcripti	0.122743923	0.911	0.025	#NUM!	CSF1R
Activation	0.125602996	0.901	0.0244	#NUM!	CARM1
Transport	0.125602996	0.901	0.0244	#NUM!	LCN1
MyD88:MA	0.128528666	0.891	0.0238	#NUM!	UBE2N
Productior	0.129121927	0.889	0.0105	#NUM!	APOM,CDC42
Aggrephag	0.131522483	0.881	0.0233	#NUM!	UBE2N
Leukocyte	0.131522483	0.881	0.0104	#NUM!	ACTN1,CDC42
TAK1-depe	0.134276496	0.872	0.0227	#NUM!	UBE2N
Retinoid m	0.134276496	0.872	0.0227	#NUM!	APOM
Coronaviru	0.137088177	0.863	0.0222	#NUM!	TUBB
Mitotic G2	0.138038426	0.86	0.0101	#NUM!	NME7,TUBB
ILK Signalir	0.14028137	0.853	0.00995	#NUM!	ACTN1,CDC42
Heme sign	0.142889396	0.845	0.0213	#NUM!	CARM1
Ephrin A Si	0.142889396	0.845	0.0213	#NUM!	CDC42
Transcripti	0.148251809	0.829	0.0204	#NUM!	USP9X
Nonhomol	0.148251809	0.829	0.0204	#NUM!	UBE2N
TP53 Regu	0.148251809	0.829	0.0204	#NUM!	CARM1
PFKFB4 Sig	0.148251809	0.829	0.0204	#NUM!	HK3
Signaling b	0.151008015	0.821	0.02	#NUM!	CDC42
Netrin-1 si	0.151008015	0.821	0.02	#NUM!	CDC42
Integrin Sig	0.152756606	0.816	0.00943	#NUM!	ACTN1,CDC42
TNFR1 Sigr	0.153815464	0.813	0.0196	#NUM!	CDC42
mTOR Sign	0.154881662	0.81	0.00935	#NUM!	CDC42,EIF4A2
HSP90 cha	0.164816239	0.783	0.0182	#NUM!	PTGES3
E3 ubiquiti	0.164816239	0.783	0.0182	#NUM!	UBE2N
NLR signali	0.167880402	0.775	0.0179	#NUM!	UBE2N

Circadian C	0.175792361	0.755	0.0169	#NUM!	CARM1
Metabolisr	0.175792361	0.755	0.0169	#NUM!	TCN1
DNA Doub	0.175792361	0.755	0.0169	#NUM!	UBE2N
SPINK1 Pa	0.178648757	0.748	0.0167	#NUM!	CPN1
Sertoli Cell	0.180301774	0.744	0.00847	#NUM!	CDC42,JUP
Semaphori	0.181134009	0.742	0.0164	#NUM!	CDC42
Cytoprotec	0.1840772	0.735	0.0161	#NUM!	CARM1
UFMylatio	0.1840772	0.735	0.0161	#NUM!	EIF4A2
Peroxisom	0.186637969	0.729	0.0159	#NUM!	USP9X
Mitochonc	0.189234362	0.723	0.0156	#NUM!	PAM16
Mitochonc	0.189234362	0.723	0.0156	#NUM!	CARM1
Actin Cyto	0.189670592	0.722	0.0082	#NUM!	ACTN1,CDC42
Neddylatic	0.192309173	0.716	0.00813	#NUM!	COPS3,DPP3
Agrin Inter	0.202301918	0.694	0.0145	#NUM!	CDC42
Sperm Mo	0.205116218	0.688	0.00778	#NUM!	CSF1R,PTK7
Stearate Bi	0.207491352	0.683	0.0141	#NUM!	BDH2
COPII-med	0.210377844	0.677	0.0139	#NUM!	CTSC
Ephrin B Si	0.210377844	0.677	0.0139	#NUM!	CDC42
Amyloid fil	0.212813905	0.672	0.0137	#NUM!	USP9X
Glioma Inv	0.212813905	0.672	0.0137	#NUM!	CDC42
Granzyme	0.215278173	0.667	0.0135	#NUM!	NME1
GDNF Fam	0.220800473	0.656	0.0132	#NUM!	CDC42
Hypoxia Si	0.220800473	0.656	0.0132	#NUM!	UBE2N
Protein Ub	0.224388192	0.649	0.00733	#NUM!	UBE2N,USP9X
Costimulat	0.225943577	0.646	0.0128	#NUM!	CDC42
VDR/RXR A	0.225943577	0.646	0.0128	#NUM!	SERPINB1
Neurotrop	0.225943577	0.646	0.0128	#NUM!	CDC42
Maturity O	0.228034207	0.642	0.0127	#NUM!	APOM
Renal Cell	0.228034207	0.642	0.0127	#NUM!	CDC42
G alpha (1	0.230674719	0.637	0.0125	#NUM!	OBSCN
Glucose m	0.230674719	0.637	0.0125	#NUM!	HK3
Transport	0.238231947	0.623	0.012	#NUM!	SLC6A14
Transcripti	0.240990543	0.618	0.0119	#NUM!	CARM1
LPS-stimul	0.243220401	0.614	0.0118	#NUM!	CDC42
Sphingolipi	0.248313311	0.605	0.0115	#NUM!	GM2A
Xenobiotic	0.248313311	0.605	0.0115	#NUM!	PTGES3
TP53 Regu	0.250610925	0.601	0.0114	#NUM!	GSR
Regulation	0.255858589	0.592	0.0111	#NUM!	ACTN1
Unfolded p	0.255858589	0.592	0.0111	#NUM!	CD82
KEAP1-NFE	0.258226019	0.588	0.011	#NUM!	DPP3
Factors inv	0.258226019	0.588	0.011	#NUM!	CDC42
ABRA Signa	0.260615355	0.584	0.0109	#NUM!	ACTN1
MAPK6/M	0.260615355	0.584	0.0109	#NUM!	CDC42
Actin Nucl	0.263026799	0.58	0.0108	#NUM!	CDC42
ERBB Signa	0.263026799	0.58	0.0108	#NUM!	CDC42
EPH-Ephrir	0.265460556	0.576	0.0106	#NUM!	CDC42
Fcγ Recept	0.265460556	0.576	0.0106	#NUM!	CDC42

Role of Ost	0.266685866	0.574	0.00649	#NUM!	CDC42,CSF1R
TGF-β Sign	0.270395836	0.568	0.0104	#NUM!	CDC42
Cellular res	0.272270131	0.565	0.0103	#NUM!	PTGES3
Glyceropho	0.27733201	0.557	0.0101	#NUM!	STARD10
VEGF Signa	0.27733201	0.557	0.0101	#NUM!	ACTN1
p75 NTR re	0.279898132	0.553	0.01	#NUM!	OBSCN
Sumoylatic	0.287078058	0.542	0.00971	#NUM!	CDC42
Transport c	0.289067988	0.539	0.00962	#NUM!	SLC6A14
Extracellula	0.293764965	0.532	0.00943	#NUM!	TNXB
Selenoami	0.295801247	0.529	0.00935	#NUM!	GSR
ISGylation	0.298538262	0.525	0.00926	#NUM!	UBE2N
Telomeras	0.298538262	0.525	0.00926	#NUM!	PTGES3
HDR throu	0.307609681	0.512	0.00893	#NUM!	UBE2N
Regulation	0.314050869	0.503	0.0087	#NUM!	CDC42
Signaling b	0.316956746	0.499	0.00862	#NUM!	CDC42
PAK Signali	0.319153786	0.496	0.00855	#NUM!	CDC42
Virus Entry	0.321366054	0.493	0.00847	#NUM!	CDC42
Regulation	0.323593657	0.49	0.0084	#NUM!	CARM1
Cholecysto	0.323593657	0.49	0.0084	#NUM!	CDC42
Sphingosin	0.325836701	0.487	0.00833	#NUM!	CDC42
NGF Signal	0.325836701	0.487	0.00833	#NUM!	CDC42
Eukaryotic	0.329609712	0.482	0.0082	#NUM!	EIF4A2
LXR/RXR A	0.331894458	0.479	0.00813	#NUM!	APOM
TCR signali	0.338844156	0.47	0.00794	#NUM!	UBE2N
MHC class	0.338844156	0.47	0.00794	#NUM!	CTSC
Pancreatic	0.338844156	0.47	0.00794	#NUM!	CDC42
ROBO SLIT	0.341192912	0.467	0.00787	#NUM!	CDC42
14-3-3-me	0.341192912	0.467	0.00787	#NUM!	TUBB
Interleukin	0.345143739	0.462	0.00775	#NUM!	UBE2N
G Beta Gar	0.345143739	0.462	0.00775	#NUM!	CDC42
Clathrin-m	0.347536161	0.459	0.00769	#NUM!	SCARB2
Class I MH	0.347536161	0.459	0.00533	#NUM!	BLMH,UBE2N
fMLP Signa	0.349140315	0.457	0.00763	#NUM!	CDC42
HGF Signal	0.351560441	0.454	0.00758	#NUM!	CDC42
Gα12/13 S	0.353997341	0.451	0.00752	#NUM!	CDC42
Atheroscle	0.353997341	0.451	0.00752	#NUM!	APOM
DHCR24 Si	0.362242998	0.441	0.0073	#NUM!	APOM
RAC Signal	0.362242998	0.441	0.0073	#NUM!	CDC42
Reelin Sign	0.364753947	0.438	0.00725	#NUM!	CDC42
RHO GTPa	0.366437575	0.436	0.00719	#NUM!	CDC42
Neutrophil	0.376703799	0.424	0.005	#NUM!	PAM16,SERPINB1
C-type lect	0.378442585	0.422	0.0069	#NUM!	UBE2N
NAD Signal	0.390840896	0.408	0.00662	#NUM!	NT5E
PTEN Signa	0.390840896	0.408	0.00662	#NUM!	CDC42
Necroptosi	0.399024902	0.399	0.00645	#NUM!	PAM16
Epithelial A	0.404575892	0.393	0.00633	#NUM!	CDC42
Aryl Hydro	0.406443329	0.391	0.00629	#NUM!	PTGES3

RAR Activa	0.410204103	0.387	0.00466	#NUM!	CARM1,CDC42
HMGB1 Sig	0.422668614	0.374	0.00599	#NUM!	CDC42
CXCR4 Sigr	0.424619564	0.372	0.00595	#NUM!	CDC42
Gαq Signal	0.427562886	0.369	0.00588	#NUM!	CDC42
Glioblastor	0.429536427	0.367	0.00585	#NUM!	CDC42
Netrin Sigr	0.433510878	0.363	0.00578	#NUM!	CDC42
Tight Junct	0.446683592	0.35	0.00556	#NUM!	CDC42
Regulation	0.453941617	0.343	0.00543	#NUM!	EIF4A2
MicroRNA	0.459198013	0.338	0.00535	#NUM!	PTGES3
Granulocyt	0.462381021	0.335	0.00529	#NUM!	CXCL17
Macrophag	0.464515275	0.333	0.00526	#NUM!	CSF1R
GNRH Sign	0.46665938	0.331	0.00524	#NUM!	CDC42
Regulation	0.467735141	0.33	0.00521	#NUM!	CDC42
Natural Kil	0.478630092	0.32	0.00505	#NUM!	CDC42
Pulmonary	0.479733449	0.319	0.00503	#NUM!	CDC42
ID1 Signali	0.483058802	0.316	0.00498	#NUM!	PTK7
Ephrin Rec	0.4852885	0.314	0.00495	#NUM!	CDC42
Intra-Golgi	0.48752849	0.312	0.00493	#NUM!	PLIN3
Cilium Asse	0.488652359	0.311	0.0049	#NUM!	TUBB
HIF1α Sign	0.496592321	0.304	0.00478	#NUM!	HK3
IL-8 Signali	0.498884487	0.302	0.00476	#NUM!	CDC42
Agranulocy	0.498884487	0.302	0.00476	#NUM!	CXCL17
Axonal Gui	0.498884487	0.302	0.00392	#NUM!	CDC42,TUBB
Keratinizat	0.505824662	0.296	0.00467	#NUM!	JUP
RHOGDI Si	0.515228645	0.288	0.00455	#NUM!	CDC42
Multiple Sc	0.517606832	0.286	0.0045	#NUM!	C8G
Thrombin S	0.522396189	0.282	0.00444	#NUM!	CDC42
HER-2 Sign	0.526017266	0.279	0.00441	#NUM!	CDC42
Role of Ost	0.527229861	0.278	0.00439	#NUM!	CSF1R
EIF2 Signal	0.530884444	0.275	0.00435	#NUM!	EIF4A2
Mitotic Me	0.538269783	0.269	0.00426	#NUM!	IST1
IL-12 Signa	0.538269783	0.269	0.00426	#NUM!	APOM
NRF2-med	0.54200089	0.266	0.00422	#NUM!	GSR
AMPK Sign	0.549540874	0.26	0.00413	#NUM!	CAB39
Role of Ost	0.552077439	0.258	0.0041	#NUM!	CTSC
Docosahex	0.561047976	0.251	0.004	#NUM!	TNFAIP2
Cardiac Hy	0.576766463	0.239	0.00383	#NUM!	CDC42
Deubiquiti	0.579428696	0.237	0.0038	#NUM!	USP9X
Signaling b	0.584790084	0.233	0.00375	#NUM!	CDC42
Circadian F	0.586138165	0.232	0.00373	#NUM!	PTGDS
Colorectal	0.59020108	0.229	0.00369	#NUM!	CDC42
Cell Cycle (	0.591561634	0.228	0.00368	#NUM!	UBE2N
Insulin Sec	0.595662144	0.225	0.00364	#NUM!	EIF4A2
Opioid Sigr	0.602559586	0.22	0.00357	#NUM!	CDC42
Eicosanoid	0.602559586	0.22	0.00357	#NUM!	PTGDS
CLEAR Sigr	0.609536897	0.215	0.00351	#NUM!	SCPEP1
Processing	0.610942025	0.214	0.00348	#NUM!	HNRNPA3

Sirtuin Sigr	0.616595002	0.21	0.00344	#NUM!	PAM16
Xenobiotic	0.620869034	0.207	0.0034	#NUM!	PTGES3
Pathogen I	1	0	0.0027	#NUM!	CXCL17
Glucocortic	1	0	0.00172	#NUM!	PTGES3
Chaperone	1	0	0.00157	#NUM!	PLIN3
Myelinatio	1	0	0.00306	#NUM!	CDC42
Mitochonc	1	0	0.00291	#NUM!	GSR
CD28 Signa	1	0	0.00193	#NUM!	CDC42
Molecular	1	0	0.00117	#NUM!	CDC42
CDC42 Sigr	1	0	0.00174	#NUM!	CDC42
Breast Can	1	0	0.00168	#NUM!	TUBB
Gap Juncti	1	0	0.00308	#NUM!	TUBB
TEC Kinase	1	0	0.00173	#NUM!	CDC42
Estrogen R	1	0	0.00244	#NUM!	CARM1
SAPK/JNK S	1	0	0.00199	#NUM!	CDC42
Serotonin I	1	0	0.00213	#NUM!	CDC42
NF-κB Sign	1	0	0.00175	#NUM!	UBE2N
G-Protein C	1	0	0.00142	#NUM!	CDC42
Phagosome	1	0	0.00144	#NUM!	CDC42
Neuroinfla	1	0	0.00315	#NUM!	CSF1R
Synaptoge	1	0	0.00317	#NUM!	CDC42
Systemic L	1	0	0.00156	#NUM!	CDC42
Hepatic Fib	1	0	0.00236	#NUM!	CDC42

64,IGHV3-7,IGHV7-81,IGKV1-9,IGKV1D-16,IGKV2-40,IGLV1-40

IGHV3-7,IGHV7-81,IGKV1-9,IGKV1D-16,IGKV2-40,IGLV1-40

64,IGHV3-7,IGHV7-81,IGKV1-9,IGKV1D-16,IGKV2-40,IGLV1-40

Supplement table 4. Diferentially expressed proteins in IPF BALF

	FC	log2(FC)	raw.pval	#NAME?	abs log2FC
APOM	31.317	4.9689	0.049224	1.3078	4.9689
SLC38A10	14.496	3.8576	7.53E-07	6.1233	3.8576
SCPEP1	0.073201	-3.772	0.00103	2.9873	3.772
GSR	0.074055	-3.7553	5.79E-08	7.237	3.7553
IGLV1-40	12.698	3.6666	2.31E-14	13.637	3.6666
STARD10	12.637	3.6595	8.99E-06	5.0464	3.6595
IGHV3-20	0.11369	-3.1368	5.56E-06	5.2546	3.1368
PLEK	8.7585	3.1307	4.98E-06	5.3024	3.1307
PGM3	8.1449	3.0259	0.023623	1.6267	3.0259
PRG2	0.12768	-2.9694	0.014278	1.8453	2.9694
IGHV1-3	0.15216	-2.7163	0.00579	2.2373	2.7163
BLMH	0.17311	-2.5302	0.001503	2.8231	2.5302
PTGDS	0.17661	-2.5014	0.002753	2.5603	2.5014
TUBB	5.6225	2.4912	0.000813	3.0902	2.4912
HK3	5.0415	2.3339	0.04335	1.363	2.3339
IGKV2-40	0.20568	-2.2815	0.001444	2.8404	2.2815
CD82	4.8529	2.2789	4.43E-05	4.3533	2.2789
PHB2	4.6666	2.2224	0.010205	1.9912	2.2224
TNXB	4.6481	2.2166	0.024586	1.6093	2.2166
COPS3	0.2156	-2.2136	0.010572	1.9758	2.2136
PAM16	0.21951	-2.1876	0.000403	3.3952	2.1876
TMC4	4.4707	2.1605	0.000902	3.0446	2.1605
NME7	0.23454	-2.0921	1.06E-05	4.9746	2.0921
IGHV3-64	0.24512	-2.0284	0.014262	1.8458	2.0284
UGP2	3.8325	1.9383	0.001498	2.8246	1.9383
LCN1	0.26574	-1.9119	0.008369	2.0773	1.9119
JUP	0.26714	-1.9043	0.015383	1.813	1.9043
NME1	3.6492	1.8676	1.06E-05	4.9729	1.8676
CXCL17	0.27664	-1.8539	0.006229	2.2056	1.8539
PGLS	0.28185	-1.827	0.009758	2.0107	1.827
NT5E	0.28801	-1.7958	0.010201	1.9913	1.7958
USP9X	3.3182	1.7304	0.010077	1.9967	1.7304
NT5C3B	3.299	1.722	2.35E-05	4.6296	1.722
CSF1R	0.30417	-1.717	1.61E-06	5.7919	1.717
HEG1	3.1093	1.6366	0.004456	2.3511	1.6366
LY6D	3.0573	1.6123	0.047787	1.3207	1.6123
PLIN3	0.33203	-1.5906	0.024835	1.6049	1.5906
UGDH	2.9417	1.5567	0.021618	1.6652	1.5567
CARM1	0.34587	-1.5317	0.001468	2.8333	1.5317
SERPINB1	2.6946	1.4301	0.048752	1.312	1.4301
ANKFY1	2.6752	1.4197	0.013227	1.8785	1.4197
BDH2	2.6693	1.4165	0.02293	1.6396	1.4165
VMO1	2.5984	1.3776	0.006541	2.1843	1.3776
DPP3	2.5553	1.3535	0.018632	1.7297	1.3535
ACTN1	2.5404	1.3451	0.002563	2.5913	1.3451

RCSD1	2.3738	1.2472	0.029199	1.5346	1.2472
TNFAIP2	2.3657	1.2422	0.043356	1.3629	1.2422
IGHV3OR1	0.42397	-1.238	0.044081	1.3558	1.238
SH3BGRL3	2.2497	1.1697	0.000236	3.6277	1.1697
SLC6A14	2.2406	1.1639	0.020312	1.6922	1.1639
PTGES3	2.2185	1.1496	0.00431	2.3656	1.1496
PAM	0.46848	-1.0939	0.015368	1.8134	1.0939
PTK7	0.46997	-1.0894	0.013162	1.8807	1.0894
CTSC	0.47054	-1.0876	0.009152	2.0385	1.0876
IGKV1-9	0.47711	-1.0676	0.002543	2.5946	1.0676
NPL	2.0934	1.0658	0.029514	1.53	1.0658
CZIB	2.08	1.0566	0.016729	1.7765	1.0566
IGHV3-13	0.48111	-1.0556	0.017827	1.7489	1.0556
GM2A	2.0632	1.0449	0.002588	2.587	1.0449
SCARB2	2.0164	1.0118	0.047089	1.3271	1.0118
TCN1	0.50094	-0.9973	0.016495	1.7827	0.9973
IGHV7-81	0.50488	-0.986	0.046341	1.334	0.986
CPN1	1.928	0.94711	0.042896	1.3676	0.94711
PEDS1-UBI	1.9105	0.93392	0.018966	1.722	0.93392
C8G	1.8984	0.92475	0.003588	2.4452	0.92475
PZP	1.8925	0.92026	0.015136	1.82	0.92026
IGHV3OR1	0.5335	-0.90643	0.003645	2.4383	0.90643
ENOSF1	1.859	0.8945	0.003003	2.5224	0.8945
CAB39	1.8176	0.86201	0.015893	1.7988	0.86201
acc_ A0A0	0.55191	-0.8575	0.002173	2.6629	0.8575
IPO7	1.7963	0.845	0.017634	1.7536	0.845
EML4	1.7477	0.80546	0.015832	1.8005	0.80546
SUSD2	0.57255	-0.80451	0.034487	1.4623	0.80451
OBSCN	1.6853	0.75299	0.020277	1.693	0.75299
MCF2L2	0.59682	-0.74464	0.013649	1.8649	0.74464
IGHV3-7	0.59759	-0.74276	0.008805	2.0553	0.74276
FCGBP	0.6024	-0.73121	0.049209	1.308	0.73121
IST1	0.60552	-0.72375	0.043213	1.3644	0.72375
UBE2N	0.6372	-0.65018	0.012188	1.9141	0.65018
HNRNPA3	0.64925	-0.62316	0.041193	1.3852	0.62316
IGKV1D-16	1.532	0.61543	0.049543	1.305	0.61543
CDC42	0.65556	-0.6092	0.008761	2.0574	0.6092
EIF4A2	0.66067	-0.59799	0.007734	2.1116	0.59799
